# From Sensing Mechanisms to System Co‐Design: Graphene‐Based Sensors and Readout Circuits

**DOI:** 10.1002/advs.77374

**Published:** 2026-08-27

**Authors:** Jinduo Zhang, Meng Chen, Guanchen Li, Ruifeng Liu, Yingxin Wang, Ziran Zhao

**Affiliations:** ^1^ Department of Engineering Physics Tsinghua University Beijing China; ^2^ National Engineering Research Center of Dangerous Articles and Explosives Detection Technologies Beijing China

**Keywords:** graphene, PS–EQ–RF framework, readout circuits, sensors

## Abstract

Graphene‐based materials combining outstanding electrical, thermal, optical and mechanical properties have demonstrated remarkable potential for high‐performance sensing across optoelectronic, molecular, mechanical, magnetic and thermal applications. However, practical graphene sensing often involves relatively small stimulus‐induced changes in diverse electrical quantities, requiring dedicated readout electronics for accurate detection and conditioning. Existing reviews rarely treat sensing mechanisms and readout architectures in a unified system co‐design context, which leaves a gap between device demonstrations and deployable systems. To bridge device transduction and circuit interfacing, a three‐level PS–EQ–RF framework is proposed, which maps physical stimuli (PS) to electrical quantities (EQ) and to readout features (RF). Guided by the framework, this review organizes graphene‐based sensors according to electrical output characteristics into four categories: voltage‐source, current‐source, resistive (RS), and capacitive (CS) sensors. Key circuit modules, representative readout topologies, and practical implementations are analyzed for each category. We further provide system‐level co‐design guidelines, including metric mapping, architecture selection, low‐noise design, and mitigation of nonidealities, and discuss the evolution, remaining bottlenecks, and future directions of graphene‐based sensing systems. This review aims to provide a systematic view of graphene‐based sensing systems and to accelerate the transition of graphene‐based sensing technologies from laboratory prototypes to practical applications.

## Graphical abstract

This review establishes a unified PS–EQ–RF framework linking graphene sensing mechanisms, native electrical outputs, and output‐specific readout circuits. Voltage‐, current‐, resistance‐, and capacitance‐type sensing systems are systematically classified to guide architecture selection, low‐noise design, system co‐design, and practical integration.

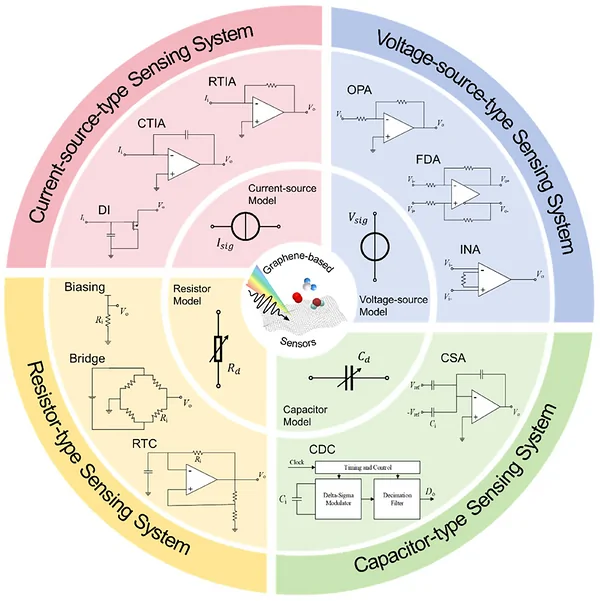

## 1 Introduction

Since the landmark achievement of mechanically exfoliating graphene by Geim and Novoselov in 2004 [1], this atomically thin carbon material has become a central topic in materials research. Researchers value graphene‐based materials for their distinctive lattice architecture and exceptional functional breadth. Prior studies have repeatedly demonstrated graphene's strengths in optical [2], electrical [3], mechanical [4, 5, 6], and thermal domains [7, 8, 9, 10]. In sensor science, the fundamental challenge lies in achieving precise conversion of external physical stimuli into reliable electronic outputs. Over the past decade, the introduction of two‐dimensional materials (2DMs) has reshaped this space by enabling sensing at nanometer‐scale platforms [11, 12, 13, 14, 15, 16]. Among them, graphene distinguishes itself as a particularly promising sensing material due to its unique combination of properties: ultrahigh electrical conductivity, exceptional carrier mobility, superior thermal conductivity, remarkable mechanical strength, and a large specific surface area [17, 18]. These coupled attributes have enabled extensive progress in graphene‐based sensing technologies across multiple applications, including photodetection [19, 20], molecular detection [21, 22, 23, 24, 25, 26, 27, 28, 29], mechanical sensing [30, 31, 32], magnetic field sensing [33, 34] and temperature sensing [35].

Despite these advances, translating device‐level demonstrations into practical systems remains challenging. Graphene‐based sensors exhibit diverse electrical output forms, and the sensing information is often encoded as stimulus‐induced electrical variations superimposed on device‐dependent baselines and noise. Therefore, the sensor‐electronics interface should not be regarded as a simple implementation issue, but rather as a defining component that affects the final signal‐to‐noise ratio (SNR), dynamic range, bandwidth, power consumption, stability, and scalability of the complete system. This issue is especially important for graphene‐based sensors because the intrinsic high surface sensitivity that enables strong responsivity also makes the devices susceptible to environmental drift [36, 37], non‐specific adsorption [38, 39], contact instability [40, 41], and device‐to‐device variability [42]. These graphene‐related nonidealities indicate that material and device optimization alone is insufficient for practical graphene sensing. Instead, readout circuits and system‐level integration should be considered together with the sensing material and device structure.

Existing reviews have provided valuable summaries of material properties, sensing mechanisms, and applications of graphene‐based sensors. However, less attention has been paid to the system‐level connection between graphene‐based sensors and the readout electronics. In particular, a clear framework that maps sensing mechanisms and native electrical output quantities to readout electronics is still lacking. This gap hinders the full realization of graphene sensors from laboratory demonstrations to practical applications.

**FIGURE 1:**
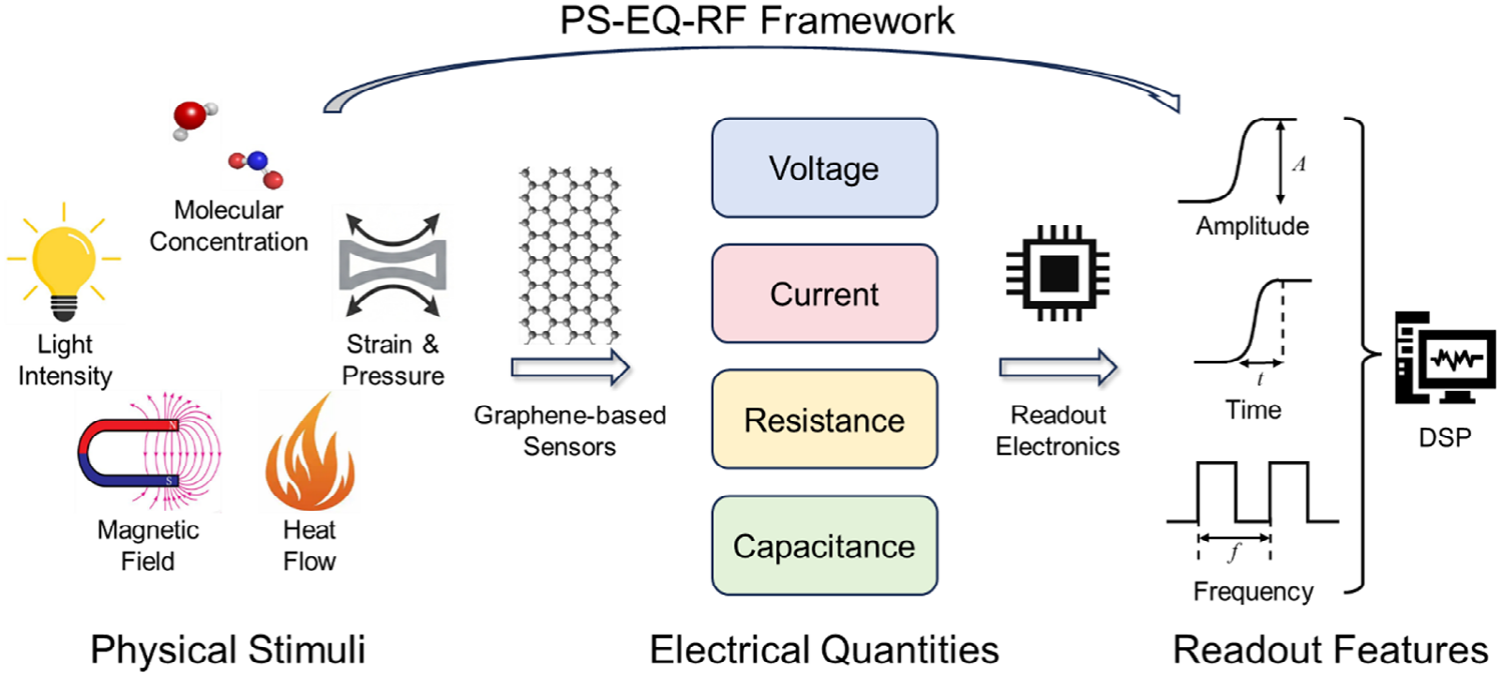
Three‐level conversion framework for graphene‐based sensors: the PS–EQ–RF framework.

**FIGURE 2:**
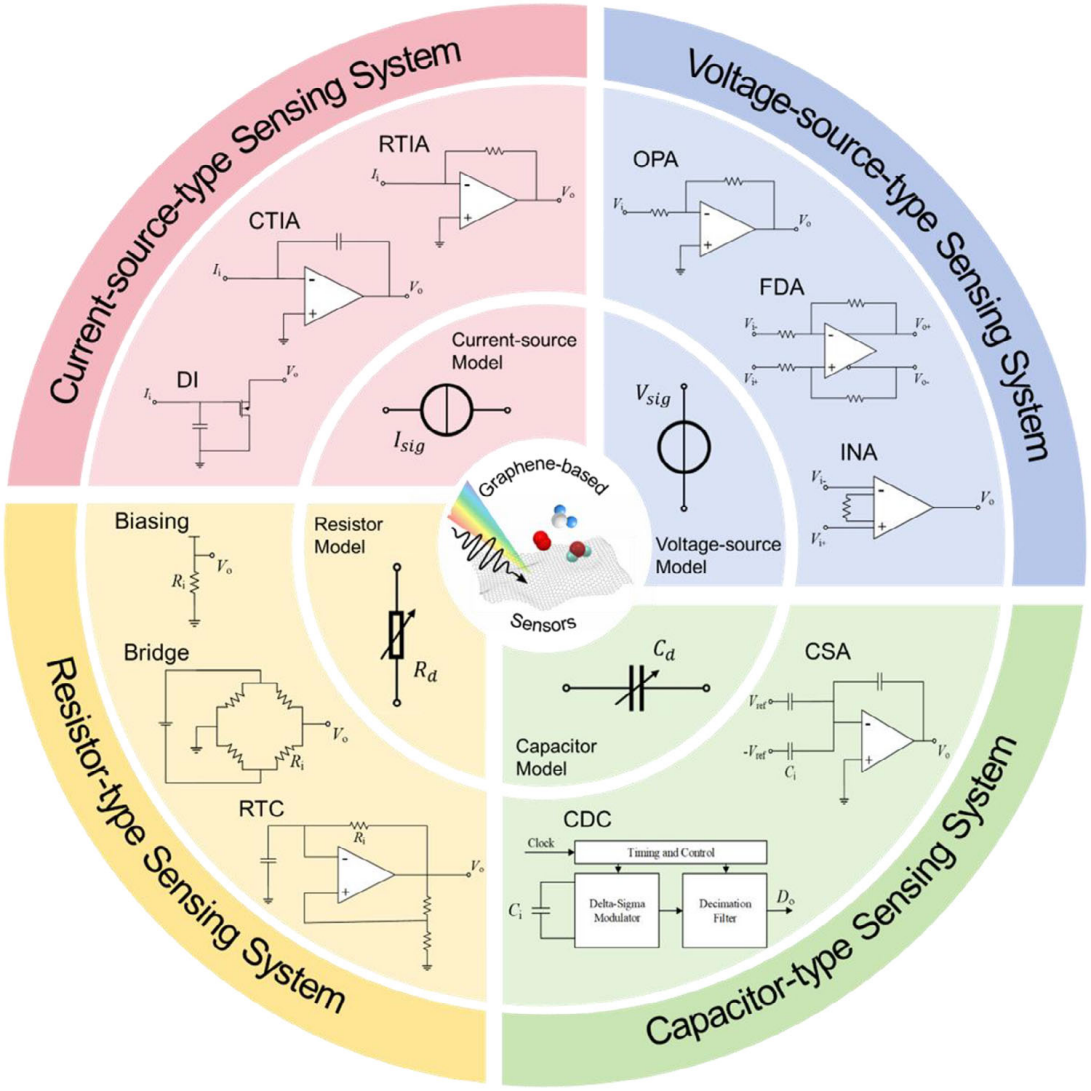
Classification of graphene‐based sensors according to four dominant electrical output characteristics with their corresponding equivalent circuit models and representative front‐end readout topologies of each category.

To address this gap, we propose a three‐level conversion framework, namely the PS–EQ–RF framework, which describes the mapping from physical stimuli (PS) to electrical quantities (EQ) and finally to readout features (RF) (Figure 1). Within this framework, the PS‐to‐EQ conversion is accomplished by the graphene‐based sensors through their transduction mechanisms, whereas the EQ‐to‐RF conversion is realized by dedicated readout circuits. Based on this framework, graphene‐based sensors are organized according to their native electrical output types (Figure 2). Representative readout topologies and implementation platforms are then discussed for each output category. We further provide system‐level design guidelines, including a co‐design workflow, metrics mapping, selection guidelines for readout architectures, low‐noise design, and mitigation of graphene sensor nonidealities. Finally, we discuss remaining bottlenecks and future directions. To establish the broader narrative of the review, we further introduce a four‐stage evolution pathway for graphene‐based sensing systems: from material properties and sensing mechanism research, through device engineering and performance optimization, to system co‐design and heterogeneous integration, and ultimately to intelligent sensing and multimodal applications (Figure 17). This pathway reflects the progressive transition of the field from demonstrating individual material and device capabilities toward developing autonomous, integrated, and application‐oriented sensing systems.

## 2 System Co‐Design Framework for Graphene‐Based Sensing Systems

To establish a system‐level framework for the co‐design of graphene‐based sensors and their readout electronics, this section first examines the graphene‐specific opportunities and challenges that shape system design. It then introduces the PS–EQ–RF framework to describe how physical stimuli are transduced into electrical quantities and subsequently transformed into measurable readout features. Finally, graphene‐based sensors are classified according to their dominant electrical output forms and corresponding equivalent‐circuit representations, thereby linking sensor types to appropriate front‐end architectures.

### 2.1 Graphene‐Based Sensors: Opportunities and Challenges

Graphene provides an unusual combination of an atomically thin and highly accessible surface, high carrier mobility, ambipolar gate‐tunable transport, broadband optical response, and mechanical flexibility [43, 44, 45, 46]. Graphene oxide (GO), reduced graphene oxide (rGO), functionalized graphene, and graphene‐based heterostructures further broaden the applicability across a wide range of sensing tasks and operating conditions. From a system perspective, these material and device characteristics are important because they also shape the native electrical outputs, operating‐point dependence, and susceptibility to environmental interference, thereby influencing subsequent readout requirements. However, the characteristics responsible for this broad sensing capability also create a set of strongly coupled challenges.

A first challenge arises from the gapless and ambipolar transport characteristics of pristine graphene. Because pristine graphene lacks an intrinsic bandgap, many transistor‐based graphene sensors operate with a non‐negligible baseline current. The sensing signal is therefore often a small change superimposed on a bias‐dependent background, placing requirements on offset handling, front‐end dynamic range, gain selection, and analog‐to‐digital converter (ADC) resolution [47]. Moreover, graphene transconductance and low‐frequency noise both vary with carrier density and operating bias. Consequently, the operating point that maximizes the responsivity does not necessarily maximize the SNR. In some GFET implementations, low‐frequency noise can be reduced by operating the sensor near the charge‐neutrality point (CNP) using an ambipolar readout scheme [48]. Thus, biasing and readout architecture are directly involved in determining the usable sensitivity and limit of detection (LOD) rather than merely recording an intrinsic device response.

A second challenge originates from the direct exposure of the conducting channel to the environment. The high surface sensitivity of graphene improves responsivity but does not intrinsically provide selectivity or stability. In many GFET‐based sensors, the graphene layer simultaneously serves as the sensing surface and the electrical transport channel. Consequently, both the target stimulus and unwanted perturbations, such as water molecules, surface contamination, and other non‐target species, can modulate the same carrier density and introduce hysteresis, baseline shifts, and cross‐sensitivity through similar electrical pathways [49, 50]. Surface functionalization can improve molecular recognition but may also alter the electrostatic characteristics of graphene [51, 52]. Because the desired response and these interfering effects can all appear as output shifts, reliable operation generally requires differential measurement, baseline correction, and appropriate calibration.

A third challenge is associated with graphene–metal contacts and fabrication‐dependent interfaces. In practical graphene sensing devices, the measured electrical response can be influenced not only by the graphene channel but also by the electrical contacts and surrounding interfaces. Variations in contact resistance and interface properties can affect the baseline characteristics and transconductance, while fluctuations in the graphene–metal contact region can also contribute to low‐frequency noise [41, 53, 54]. Consequently, contact‐ and fabrication‐related variations can lead to differences in baseline, transconductance, and noise across nominally identical devices. From a system perspective, these variations impose additional requirements on the design of dedicated readout configurations.

A fourth challenge arises when individual graphene devices are scaled into sensor arrays. Variations in contact resistance, CNP position, transconductance, and noise become array‐level problems involving inter‐channel variability and data reliability. A single global bias condition may therefore be unsuitable for all sensing channels. Integrated graphene transistor arrays have addressed these variations using sensor redundancy, large‐scale statistical characterization, and profile‐matching calibration techniques [55, 56]. The expected distribution of sensor characteristics should be considered together with front‐end dynamic range, multiplexing strategy, channel‐specific calibration, and data processing rather than treating the sensor array and readout electronics as independent design stages.

In summary, the technological maturity of graphene‐based sensing is uneven across device and system levels. Fundamental mechanisms and single‐device characterization have been extensively studied. However, several challenges remain less mature at the system level, including bias‐ and noise‐dependent operation arising from graphene's gapless and ambipolar transport, environmental interference, contact‐ and fabrication‐induced variability, and array‐level nonuniformity, all of which increase the requirements for calibration, channel management, and coordinated readout design. The practical performance of the graphene‐based sensing system is jointly determined by the device structure, interfaces, noise, front‐end dynamic range, calibration strategies, and array architecture. These coupled dependencies motivate the introduction of the PS–EQ–RF framework.

### 2.2 PS–EQ–RF Framework

The PS–EQ–RF framework describes the mapping from physical stimuli (PS) to electrical quantities (EQ) and finally to readout features (RF), as illustrated in Figure 1. Within this framework, the PS‐to‐EQ conversion corresponds to the sensing process. Depending on the application, the typical physical stimuli may include light intensity, molecular concentration, strain and pressure, magnetic field, and heat flow. Graphene‐based sensors convert these stimuli into electrical quantities through multiple mechanisms, as discussed in Section 3. The resulting electrical quantities may appear as a generated voltage or current, or a stimulus‐dependent resistance or capacitance, whose variations encode the magnitude or state of the physical stimulus. The EQ‐to‐RF conversion corresponds to the electronic readout process. The core design paradigm emphasizes transforming sensor‐side electrical quantities that contain the target information but may not be directly measurable, into robust readout features that can be readily conditioned and converted by standard ADCs. These readout features convey sensing information through amplitude‐domain, time‐domain, or frequency‐domain characteristics. By distinguishing the physical transduction stage from the electronic feature‐extraction stage while retaining their mutual dependence, the PS–EQ–RF framework provides a common basis for comparing different graphene sensing systems and co‐optimizing their devices and readout circuits.

### 2.3 Classification by Electrical Output Characteristics

From an application standpoint, researchers commonly group graphene‐based sensors into several functional categories. Photoelectric sensors are employed for light detection, molecular sensors are designed for chemical or biomolecular recognition, mechanical sensors are used for strain and pressure monitoring, magnetic sensors enable the detection of magnetic fields, and thermal sensors are applied to temperature measurement. However, an application‐oriented classification alone does not specify the electrical interface presented to the readout circuit. Graphene‐based sensors can therefore also be classified according to their dominant electrical output characteristics, as shown in Figure 2. Voltage‐source‐type sensors (VSS) generate output signals that can be represented by a Thévenin‐type voltage source model, whereas current‐source‐type sensors (CSS) are more naturally modeled by a Norton‐type current source. Other categories include resistor‐type sensors (RS), where stimulus‐induced changes appear as resistance modulation that can be captured by a variable‐resistor model, and capacitor‐type sensors (CS), where the stimulus primarily modulates capacitance and is described by a variable‐capacitor model. This complementary application‐ and interface‐oriented classification, which links application scenarios to signal‐output mechanisms, provides a practical framework for sensor optimization and readout co‐design. The framework helps researchers map sensing targets to device architectures, and it also helps circuit designers select appropriate front‐end topologies that match the sensor's output form and signal scale (Figure 2).

## 3 Graphene‐Based Sensors

**FIGURE 3:**
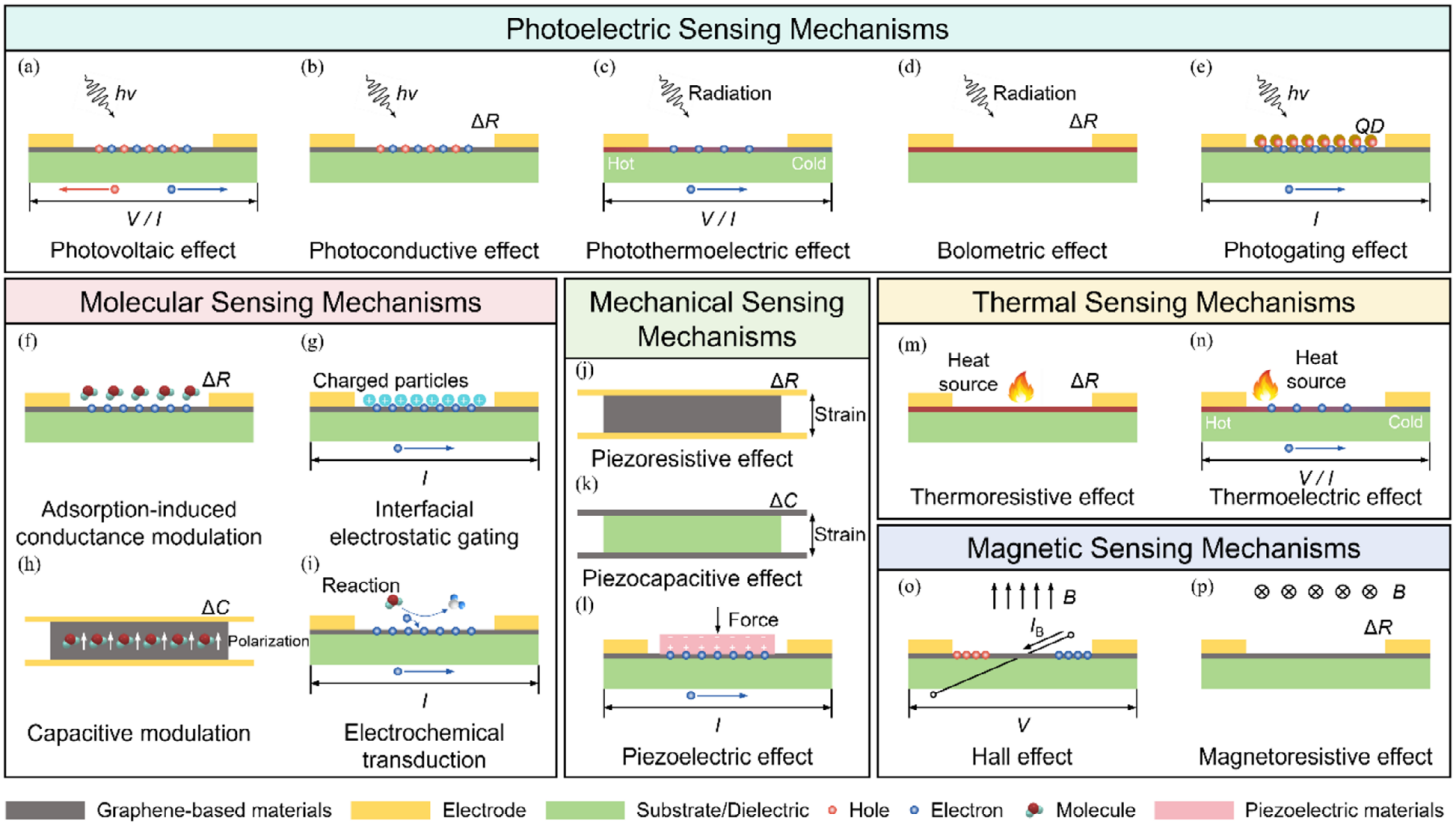
Schematic illustrations of the principal mechanisms in graphene‐based sensors. (a–e) Photoelectric sensing mechanisms: photovoltaic, photoconductive, photothermoelectric, bolometric, and photogating effects; (f–i) Molecular sensing mechanisms: adsorption‐induced conductance modulation, interfacial electrostatic gating, capacitive modulation, and electrochemical transduction. (j–l) Mechanical sensing mechanisms: piezoresistive, piezocapacitive, and piezoelectric effects; (m,n) Thermal sensing mechanisms: thermoresistive and thermoelectric effects. (o,p) Magnetic sensing mechanisms: Hall and magnetoresistive effects. QD: quantum dots; *I*_B_: bias current.

To connect the PS‐to‐EQ conversion introduced in Section 2 with practical graphene‐based sensor implementations, this section systematically examines photoelectric, molecular, mechanical, thermal, and magnetic sensing modalities. Figure 3 illustrates the principal sensing mechanisms, while Table 1 summarizes the corresponding input–output relationships, electrical output types, and fundamental performance metrics. These metrics are selected to capture five basic aspects of sensing performance: the magnitude of the sensing response, the minimum detectable input, the usable measurement range, the response speed, and the fidelity of the sensing response. The following subsections further discuss the operating principles and representative device implementations for each sensing modality.

**TABLE 1:** Mechanisms, input–output relationships, electrical output types, and fundamental performance metrics of graphene‐based sensors across different sensing modalities. *P*: optical power; *c*: concentration or activity; *X*: mechanical input; *T*: temperature; and *B*: magnetic flux density.

Sensing modality	Mechanism	Input–output relationship	Electrical output type	Fundamental performance metrics
Photoelectric sensor	Photovoltaic effect	*P* → *V*/*I*	VSS/CSS	Responsivity (*R_V_* or *R_I_*); NEP, specific detectivity (D*); LDR, spectral response range; response time and bandwidth; linearity
	Photoconductive effect	*P* → Δ*R*	RS	
	Photothermoelectric effect	*P* → *V*/*I*	VSS/CSS	
	Bolometric effect	*P* → Δ*R*	RS	
	Photogating effect	*P* → *I*	CSS	
Molecular sensor	Adsorption‐induced conductance modulation	*c* → Δ*R*	RS	Sensitivity; LOD; working range; response and recovery times; selectivity
	Interfacial electrostatic gating	*c* → *I*	CSS	
	Capacitive modulation	*c* → Δ*C*	CS	
	Electrochemical transduction	*c* → *I*	CSS	
Mechanical sensor	Piezoresistive effect	*X* → Δ*R*	RS	Sensitivity, gauge factor; LOD; working range; response and recovery times; linearity
	Piezocapacitive effect	*X* → Δ*C*	CS	
	Piezoelectric effect	*X* → *V*/*I*	VSS/CSS	
Thermal sensor	Thermoresistive effect	*T* → *R*	RS	TCR, Seebeck coefficient; temperature resolution; working range; response and recovery times; linearity
	Thermoelectric effect	Δ*T* → *V*/*I*	VSS/CSS	
Magnetic sensor	Hall effect	*B* → *V*	VSS	Hall sensitivity, magnetoresistance ratio; noise‐equivalent magnetic field; working range; response time and bandwidth; linearity
	Magnetoresistive effect	*B* → Δ*R*	RS	

### 3.1 Graphene‐Based Photoelectric Sensors

**FIGURE 4:**
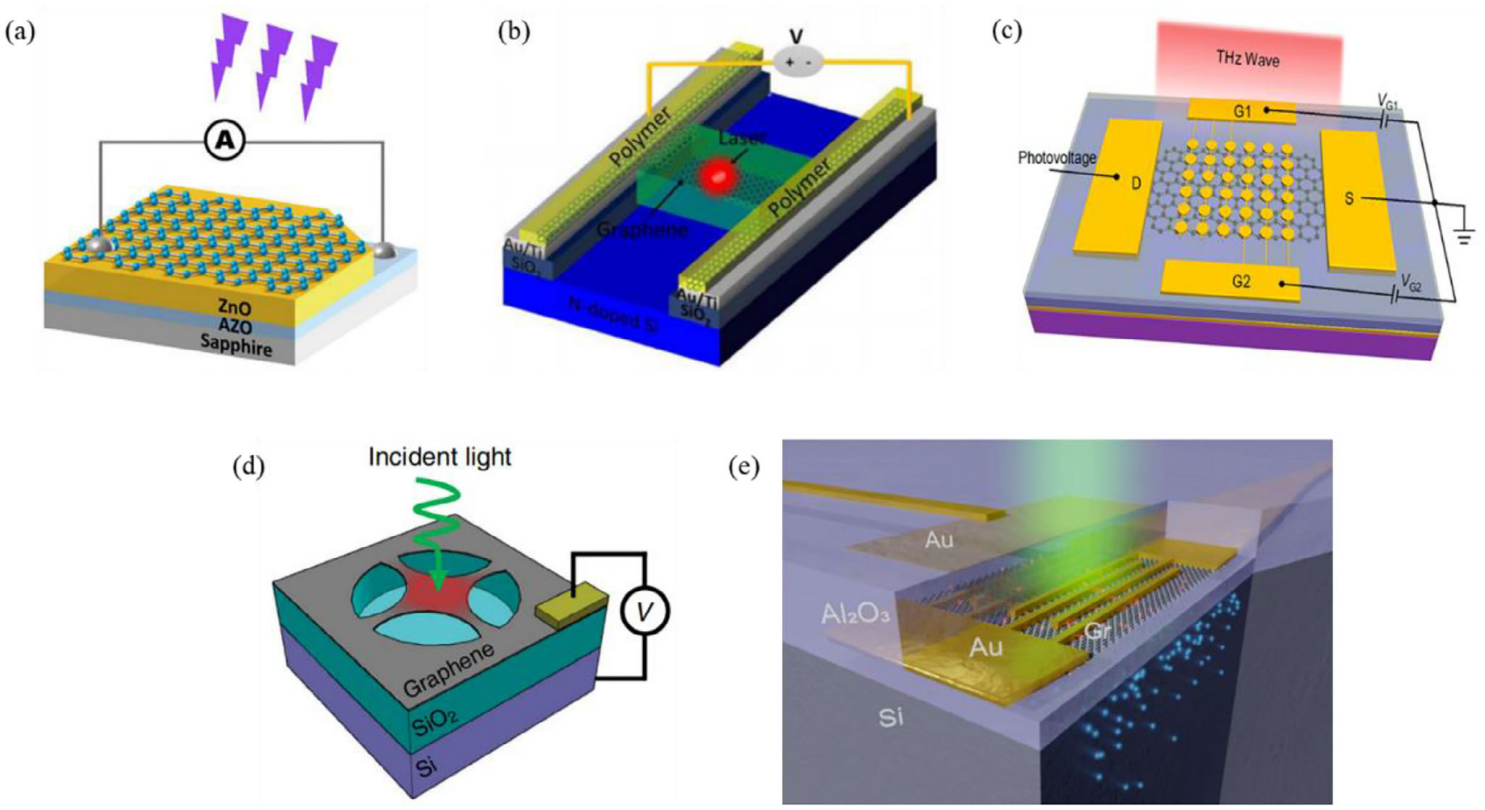
Representative device architectures of graphene‐based photoelectric sensors, illustrating five operating mechanisms: (a) PV sensor. Reproduced with permission [59]. Copyright 2020, Elsevier; (b) PC sensor. Reproduced under terms of the CC‐BY license [57]. Copyright 2017, published by Springer Nature; (c) PTE sensor. Reproduced with permission [60]. Copyright 2022, American Chemical Society; (d) Bolometric sensor. Reproduced under terms of the CC‐BY license [58]. Copyright 2019, published by Springer Nature; (e) Photogating sensor. Reproduced under terms of the CC‐BY license [61]. Copyright 2024, published by American Chemical Society.

Graphene‐based photoelectric sensors convert incident optical radiation into measurable electrical quantities. Typical operating mechanisms include photovoltaic (PV), photoconductive (PC), photothermoelectric (PTE), bolometric, and photogating effects. Their operating principles are illustrated in Figure 3a–e, while representative implementations based on these mechanisms are shown in Figure 4 [57, 58, 59, 60, 61].

A PV response arises when photoexcited carriers are separated by an internal electric field associated with a junction [62, 63]. In a simplified carrier‐collection model, the short‐circuit photocurrent can be expressed as:

(1) $$I_{\mathrm{PV}}=e\eta_{\mathrm{col}}\Phi_{\mathrm{abs}}$$

where *e* is the elementary charge, η_col_ is the carrier‐collection efficiency, and Φ_abs_ is the absorbed photon rate. Depending on the electrical boundary condition, the same junction may be characterized by a short‐circuit photocurrent or an open‐circuit photovoltage. PV sensors can therefore be classified as VSS or CSS.

The PC mechanism originates from an illumination‐induced change in channel conductance [64]. The primary conductance variation Δ*G*_PC_ can be expressed as:

(2) $$\Delta G_{\mathrm{PC}}=G_{\mathrm{ch}}^{\mathrm{light}}\;-\;G_{\mathrm{ch}}^{\mathrm{dark}}$$

where $G_{\mathrm{ch}}^{\mathrm{light}}$ and $G_{\mathrm{ch}}^{\mathrm{dark}}$ are the channel conductances under illumination and in the dark, respectively. Equivalently, the response may be represented by the corresponding resistance change Δ*R*_PC_. PC sensors can therefore be classified as RS.

The PTE effect results from nonuniform heating of the electronic system [65, 66, 67, 68]. When optical absorption produces an electron‐temperature gradient, and the Seebeck coefficient varies spatially along the graphene channel, the thermoelectric voltage measured between positions *a* and *b* can be written:

(3) $$V_{\mathrm{PTE}}=-\int_{a}^{b}S\left(x\right)\frac{dT_{e}\left(x\right)}{dx}dx$$

where *S*(*x*) is the local Seebeck coefficient and *T*_e_(*x*) is the local electron temperature. In the PTE mechanism, the directly generated quantity is a thermoelectric voltage, although a photocurrent may also be measured when the device is connected to a finite external load. Therefore, PTE sensors can be classified as VSS or CSS.

The bolometric effect begins with optical heating, and the measurable quantity is the resulting change of the temperature‐dependent resistance [63, 68, 69]. The primary electrical variation is the channel resistance change, which can be expressed as:

(4) $$\Delta R_{\mathrm{bol}}\;\approx \;{\left.\frac{dR_{\mathrm{ch}}}{dT}\right|}_{T_{0}}\Delta T$$

where *R*_ch_ is the channel resistance, *T*_0_ is the operating temperature, and Δ*T* is the optically induced temperature change. Graphene bolometers are therefore classified as RS.

In photogating devices, optical excitation induces a relatively long‐lived effective charge near the graphene channel. Depending on the device structure, this effective charge may originate from carrier trapping or transfer in an adjacent absorber, substrate, or the interface [70, 71]. The resulting electrostatic perturbation acts as an effective gate‐voltage perturbation Δ*V*_PG_ with a magnitude of |Δ*Q*_eff_|/*C*_eff_, where Δ*Q*_eff_ is the trapped charge and *C*_eff_ is the effective capacitance. For a sufficiently small perturbation around a fixed operating point, the resulting drain‐current variation is:

(5) $$\Delta I_{\mathrm{PG}}\;\approx \;g_{m}\Delta V_{\mathrm{PG}}$$

where *g*_m_ is the transconductance. Photogating is therefore more accurately interpreted as a photoinduced, gate‐modulated CSS.

The fundamental performance metrics for graphene‐based photoelectric sensors include voltage responsivity *R_V_* or current responsivity *R_I_*, noise‐equivalent power (NEP), specific detectivity D*, linear dynamic range (LDR), spectral response range, response time and bandwidth, and linearity [72, 73, 74], as summarized in Table 1. Responsivity quantifies the output current or voltage generated per unit incident optical power. NEP is the incident optical power required to produce a signal equal to the output noise within a 1‐Hz bandwidth, while specific detectivity normalizes NEP with respect to the effective photosensitive area *A* according to $\sqrt{A}/\mathrm{NEP}$ when NEP is expressed in W/Hz^1/2^. LDR defines the range of incident optical power over which the sensor output remains approximately proportional to the input. Spectral response range defines the wavelength interval over which the detector provides a measurable response. Response time and bandwidth characterize the temporal performance of a sensor. Linearity characterizes how closely the sensor response follows a linear input‐output relationship over the specified measurement range.

### 3.2 Graphene‐Based Molecular Sensors

**FIGURE 5:**
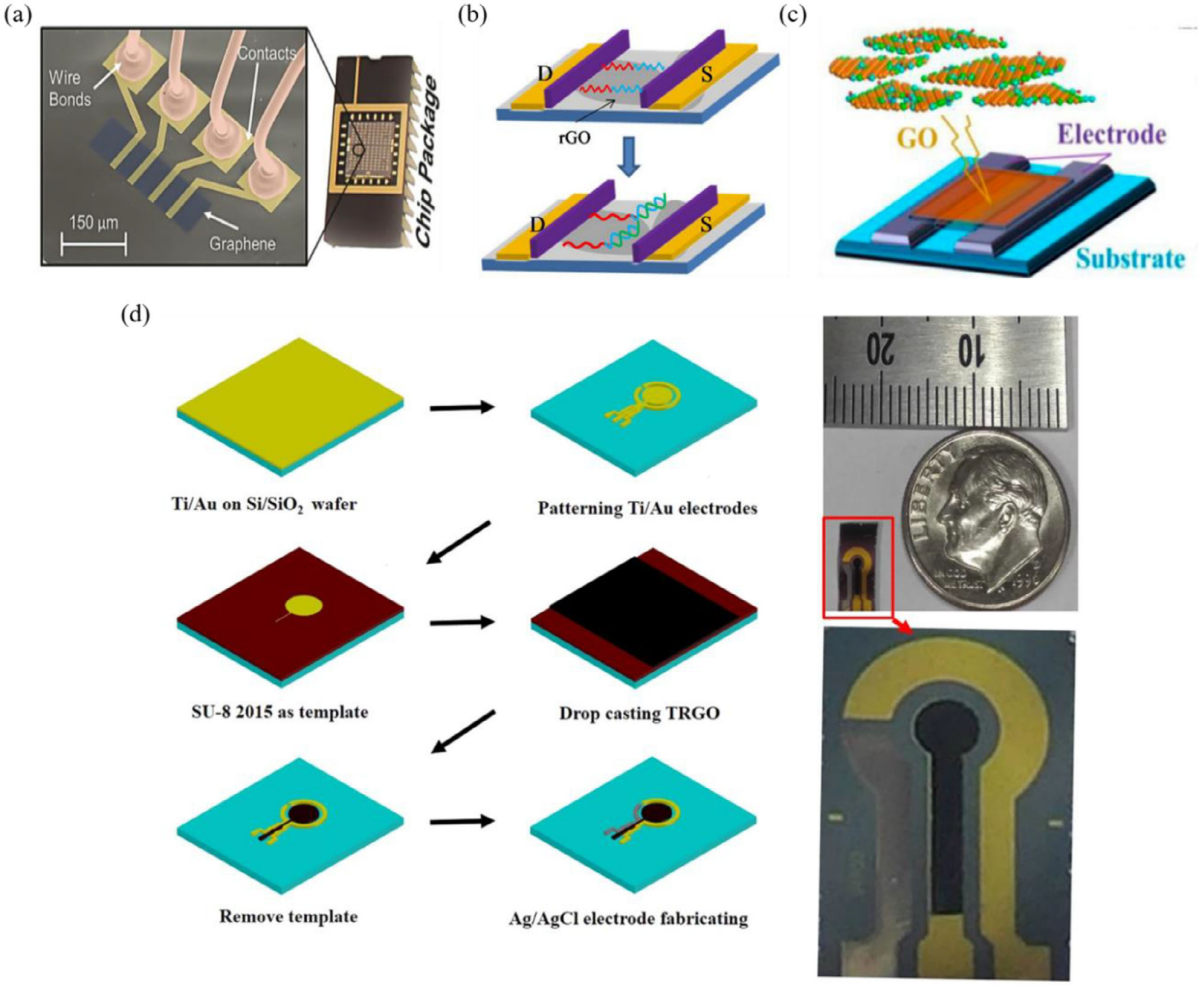
Representative device architectures of graphene‐based molecular sensors, illustrating four operating mechanisms: (a) Adsorption‐induced conductance modulation sensor. Reproduced under terms of the CC‐BY license [82]. Copyright 2015, published by Royal Society of Chemistry; (b) Interfacial electrostatic gating sensor. Reproduced with permission [81]. Copyright 2017, Elsevier; (c) Capacitive modulation sensor. Reproduced with permission [80]. Copyright 2018, American Chemical Society; (d) Electrochemical transduction sensor. Reproduced under terms of the CC‐BY license [79]. Copyright 2016, published by Springer Nature.

Graphene‐based molecular sensors convert analyte concentration, gas partial pressure, chemical activity, or molecular‐binding events into electrical signals [75, 76]. Pristine graphene offers an atomically thin, directly exposed conductive surface for interactions with adsorbates, while defects, grain boundaries, and oxygen‐containing functionalities in GO and rGO can introduce additional adsorption or functionalization sites, and intentionally introduced receptors can provide target‐specific molecular recognition [77, 78]. Typical operating mechanisms include adsorption‐induced conductance modulation, interfacial electrostatic gating, capacitive modulation, and electrochemical transduction. The corresponding operating principles are illustrated in Figure 3f–i, while representative device implementations based on these mechanisms are shown in Figure 5 [79, 80, 81, 82].

In adsorption‐induced conductance modulation, adsorbed species may donate or withdraw charge or act as charged scattering centers, thereby altering the carrier density, carrier mobility, or both, and hence the channel conductance [28, 43, 83]. For a spatially uniform graphene channel neglecting contact resistance, a simplified two‐carrier conductance model is:

(6) $$G_{\mathrm{ch}}\;\approx \;\frac{W}{L}e\left(\mu_{e}n+\mu_{h}p\right)$$

where *W* is the channel width, *L* is the channel length, *n* and *p* are the electron and hole sheet densities, and µ_e_ and µ_h_ are their mobilities, respectively. Molecular sensors based on adsorption‐induced conductance modulation can therefore be classified as RS.

In interfacial electrostatic gating mechanisms, adsorbed molecules, the ionization of surface functional groups, or the binding of charged biomolecules changes the effective surface potential, thereby shifting the graphene transfer characteristic, typically shown as the change of the drain current at a fixed bias [84, 85, 86]. For a small perturbation around a fixed operating point, the resulting drain current can be expressed in a form analogous to Equation (5), with the molecular interaction represented as an effective gate‐potential variation. Molecular sensors based on interfacial electrostatic gating can therefore be classified as a molecule‐induced, gate‐modulated CSS.

In capacitive modulation, polar molecules adsorbed by GO or other graphene‐based dielectric layers can modify their effective dielectric properties [87]. Specifically, molecular adsorption may consequently alter the capacitance through changes in the effective dielectric permittivity and, in deformable structures, through changes in the effective geometry. In an idealized parallel plate representation, we have:

(7) $$C\;\approx \;\frac{\varepsilon_{0}\varepsilon_{\mathrm{eff}}A}{d}$$

where *A* is the effective electrode area, *d* is the effective electrode spacing, ε_0_ is the permittivity of vacuum, and ε_eff_ is the effective relative permittivity. Molecular sensors based on capacitive modulation can therefore be classified as CS.

In electrochemical transduction, graphene‐based materials act as electrodes, while oxidation, reduction, or other interfacial charge transfer reactions produce a faradaic current *I*_F_ [88, 89, 90]. If *J* is the molar flux of an electroactive species at the electrode surface, Faraday's law gives:

(8) $$I_{F}=nFAJ$$

where *n* is the electron transfer number and *F* is the Faraday constant. Molecular sensors based on electrochemical transduction can therefore be classified as CSS.

The fundamental performance metrics for graphene‐based molecular sensors include sensitivity, LOD, working range, response and recovery times, and selectivity [43, 91], as summarized in Table 1. Sensitivity quantifies the change in sensor output per unit change in the input quantity. LOD defines the lowest analyte level that can be statistically distinguished from the blank under specified measurement conditions. Working range defines the input interval over which the sensor provides a reliable response. Response and recovery times characterize the temporal processes by which the sensor approaches and returns from its analyte‐induced response. Selectivity characterizes the ability of a sensor to distinguish the target analyte from relevant interferents.

### 3.3 Graphene‐Based Mechanical Sensors

**FIGURE 6:**
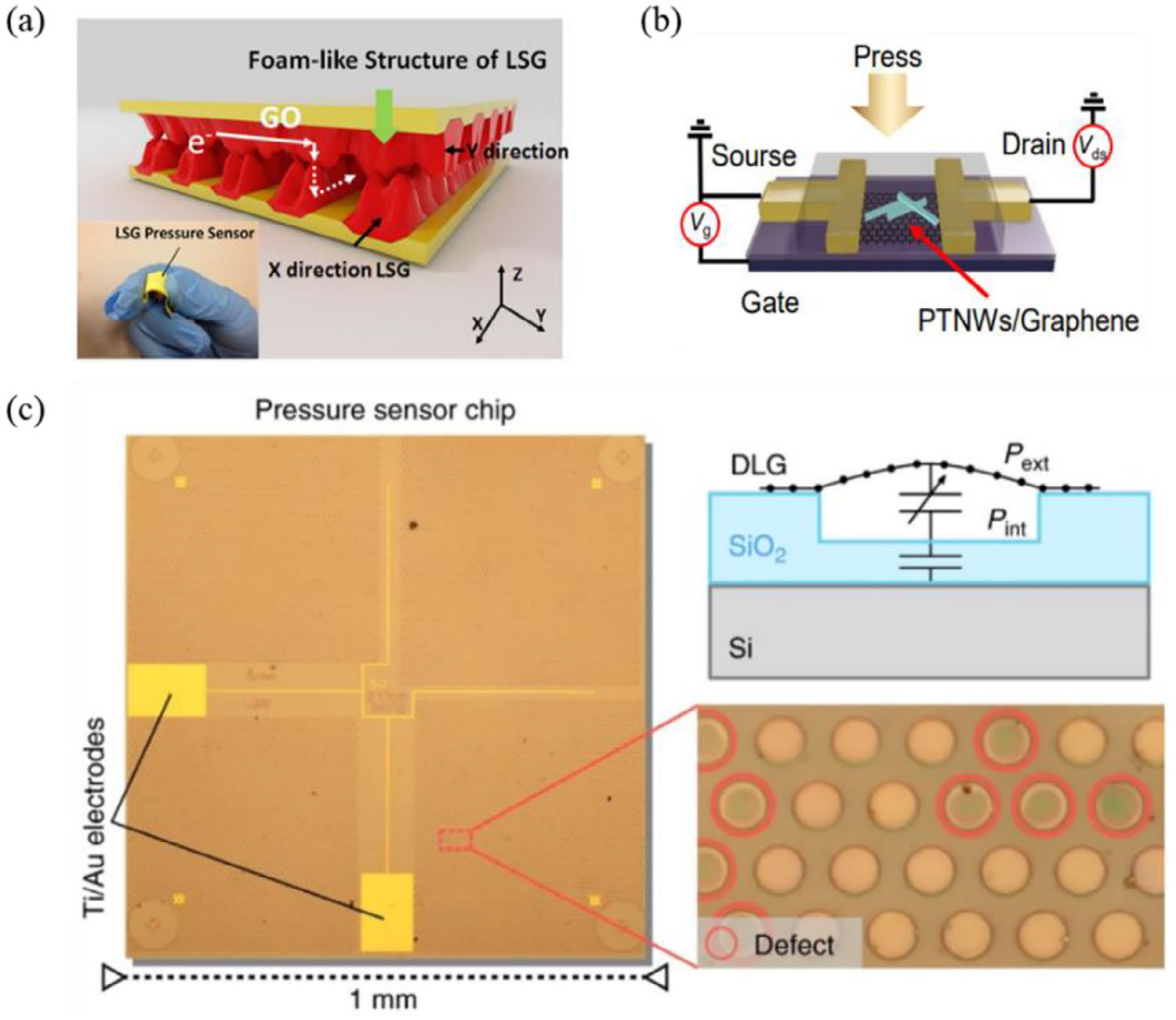
Representative device architectures of graphene‐based mechanical sensors, illustrating three operating mechanisms. (a) Piezoresistive sensor. Reproduced under terms of the CC‐BY license [92]. Copyright 2015, published by Springer Nature; (b) Piezoelectric sensor. Reproduced with permission [93]. Copyright 2017, American Chemical Society; (c) Piezocapacitive sensor. Reproduced under terms of the CC‐BY license [94]. Copyright 2020, published by Springer Nature.

Graphene‐based mechanical sensors convert mechanical stimuli such as pressure, strain, force, and vibration into electrical signals through deformation‐induced modifications of their electrical properties. Typical operating mechanisms include the piezoresistive, piezocapacitive, and piezoelectric effects. The corresponding operating principles are illustrated in Figure 3j–l, while representative device implementations based on these mechanisms are shown in Figure 6 [92, 93, 94].

Piezoresistive sensors operate through deformation‐induced changes in electrical resistance [95, 96, 97, 98, 99]. Mechanical deformation can change both the channel geometry and the sheet resistance *R*_s_, with the latter arising from strain‐dependent changes in carrier density, mobility, and band structure. For a homogeneous rectangular two‐dimensional conductor, the resistance *R* can be expressed as:

(9) $$R=R_{s}\;\frac{L}{W}$$

Piezoresistive sensors can therefore be classified as RS.

Piezocapacitive sensors use graphene as a deformable membrane electrode of a capacitive structure. Mechanical deformation can change the electrode spacing, overlap area, effective dielectric permittivity, or a combination of these quantities [100]. Under an idealized parallel plate approximation, the resulting capacitance can be described by Equation (7). Piezocapacitive sensors can therefore be classified as CS.

Piezoelectric sensors operate through mechanically induced electrical polarization in a piezoelectric component [101, 102, 103, 104, 105, 106]. Mechanical deformation generates polarization charge in the piezoelectric material, and the resulting charge may either be collected through graphene electrodes or produce a piezopotential that modulates carrier transport in a graphene channel. For a conventional piezoelectric material operated in the longitudinal mode, the generated charge can be approximated as:

(10) $$Q_{\mathrm{pe}}=d_{33}\;f$$

where *d*_33_ is the longitudinal piezoelectric coefficient and *f* is the applied force. Similar to PV, the generated charge may appear as an open‐circuit voltage or a short‐circuit current. Piezoelectric sensors can therefore be classified as VSS or CSS.

The fundamental performance metrics for graphene‐based mechanical sensors include sensitivity, gauge factor, LOD, working range, response and recovery times, and linearity [107, 108], as summarized in Table 1. Among these metrics, gauge factor quantifies the relative resistance change per unit applied strain and is primarily applicable to piezoresistive strain sensors.

### 3.4 Graphene‐Based Thermal Sensors

**FIGURE 7:**
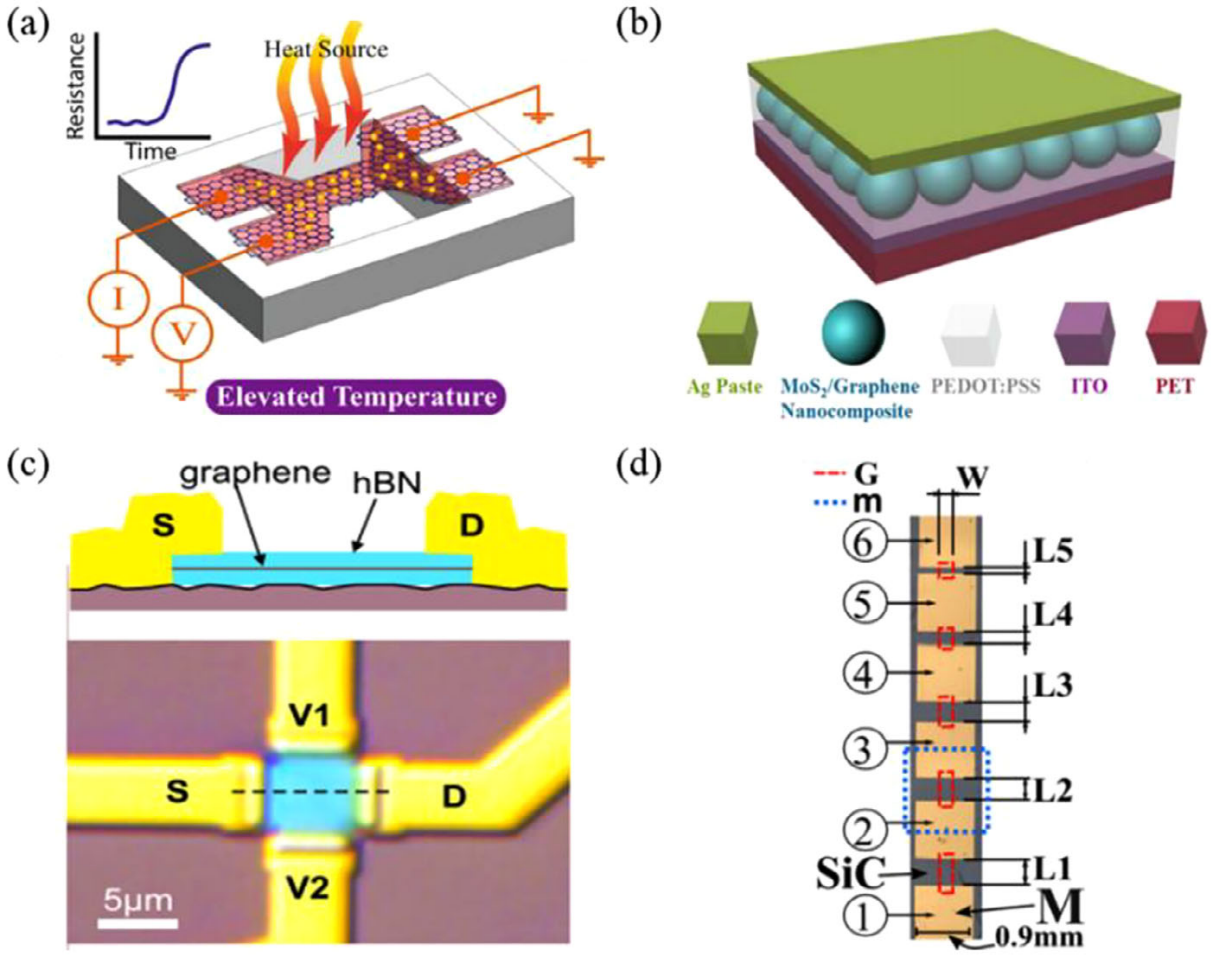
Representative device architectures of graphene‐based thermal and magnetic sensors, illustrating different operating mechanisms: (a) Thermoresistive effect sensor. Reproduced under terms of the CC‐BY license [109]. Copyright 2017, published by Springer Nature; (b) Thermoelectric effect sensor. Reproduced with permission [110]. Copyright 2017, IOP Publishing; (c) Hall effect sensor. Reproduced with permission [118]. Copyright 2015, AIP Publishing; (d) Magnetoresistive effect sensor. Reproduced with permission [119]. Copyright 2017, AIP Publishing.

Graphene‐based thermal sensors convert temperature *T* or a temperature difference Δ*T* into electrical signals through temperature‐dependent electrical transport or thermoelectric conversion. Typical operating mechanisms include the thermoresistive and thermoelectric effects. The corresponding operating principles are illustrated in Figure 3m,n, while representative device implementations based on these mechanisms are shown in Figure 7a,b [109, 110].

The thermoresistive mechanism originates from a temperature‐induced change in the electrical resistance of the sensing material [111, 112, 113]. Over a limited calibrated temperature range, the resistance variation versus temperature relationship can be expressed in the same form as Equation (4). Thermoresistive sensors can therefore be classified as RS.

The thermoelectric mechanism originates from the Seebeck effect, in which a temperature gradient produces an electromotive force [114, 115, 116, 117]. For a thermocouple consisting of two regions M and N with different Seebeck coefficients, the thermoelectric voltage can be written as:

(11) $$V_{\mathrm{TE}}=\int_{T_{\mathrm{cold}}}^{T_{\mathrm{hot}}}\;\left[S_{N}\;\left(T\right)\;-\;S_{M}\left(T\right)\right]dT$$

where *S*_M_(*T*) and *S*_N_(*T*) are the temperature‐dependent Seebeck coefficients of the two regions, and *T*_cold_ and *T*_hot_ are the temperatures at the two junctions. The directly generated quantity is an open‐circuit voltage, although a current can flow when the device is connected to a finite load. Thermoelectric sensors can therefore be classified as VSS or CSS.

The fundamental performance metrics for graphene‐based thermal sensors include the temperature coefficient of resistance (TCR), the Seebeck coefficient, temperature resolution, working range, response and recovery times, and linearity [111, 117], as summarized in Table 1. TCR quantifies the relative resistance change per unit temperature change and is applicable to thermoresistive sensors. The Seebeck coefficient quantifies the generated voltage per unit temperature difference and is applicable to thermoelectric sensors. Temperature resolution denotes the smallest temperature change that can be distinguished from the output noise.

### 3.5 Graphene‐Based Magnetic Sensors

Graphene‐based magnetic sensors receive magnetic flux density *B* as their principal input, and convert it into electrical signals. Typical operating mechanisms include the Hall and magnetoresistive effects. The corresponding operating principles are illustrated in Figure 3o,p, while representative device implementations based on these mechanisms are shown in Figure 7c,d [118, 119].

In a Hall sensor, a perpendicular magnetic field deflects moving charge carriers through the Lorentz force, producing a transverse Hall electric field and Hall voltage [46, 120]. Under a single‐carrier approximation sufficiently far from the CNP, the Hall voltage can be expressed as:

(12) $$V_{H}\;\approx \;G_{H}\frac{I_{B}B}{en_{s}}$$

where *I*_B_ is the bias current, *n*_s_ is the sheet carrier density, and *G*_H_ is a geometry‐dependent correction factor. Hall sensors can therefore be classified as VSS.

Magnetoresistive sensors convert a magnetic field into a change in longitudinal electrical resistance [34, 121]. Depending on the device structure, the magnetic‐field‐dependent resistance may arise from Lorentz‐force‐induced carrier deflection, simultaneous electron–hole transport, carrier‐density inhomogeneity, or geometry‐enhanced redistribution of current paths. The resistance variation Δ*R*_MR_ can be written as:

(13) $$\Delta R_{\mathrm{MR}}=R\left(B\right)\;-\;R\left(0\right)$$

Magnetoresistive sensors can therefore be classified as RS.

The fundamental performance metrics for graphene‐based magnetic sensors include Hall sensitivity, magnetoresistance ratio, noise‐equivalent magnetic field, working range, response time and bandwidth, and linearity [33, 122, 123], as summarized in Table 1. Hall sensitivity describes the slope of the Hall‐voltage response with magnetic field. Magnetoresistance ratio quantifies the relative change in resistance induced by a magnetic field. Noise‐equivalent magnetic field represents the input magnetic‐field noise obtained by referring the output noise through the sensor sensitivity.

## 4 Readout Methods for Graphene‐Based Sensors

After the PS‐to‐EQ conversion discussed in Section 2, readout electronics perform the second conversion in the PS–EQ–RF framework, namely the transformation from native electrical quantities to robust readout features. This conversion is important because native sensor outputs may not be directly compatible with the selected acquisition interface, and the sensing information may be encoded as relatively small variations superimposed on baseline and noise.

In practical systems, the readout chain usually needs to accomplish three coupled tasks. First, it converts the native electrical quantity into a measurable signal form compatible with downstream electronics. Since most ADCs are designed to accept voltage inputs, signals from CSS, RS, and CS devices are often converted into voltage before digitization, although time‐, frequency‐, and direct digital‐domain conversion approaches are also available. Second, the converted signal must be amplified, filtered, stabilized, and calibrated to preserve SNR, dynamic range, and linearity. Third, the conditioned signal is transformed into a readout feature that can be used for data acquisition and digital processing. These readout features may be encoded as amplitude, temporal, or frequency‐domain characteristics, and the choice depends on the sensing target and the dominant noise sources. Time‐domain processing often emphasizes amplitude tracking and transient dynamics, whereas frequency‐domain processing can improve immunity to low‐frequency noise and enable modulation‐based sensing.

**FIGURE 8:**
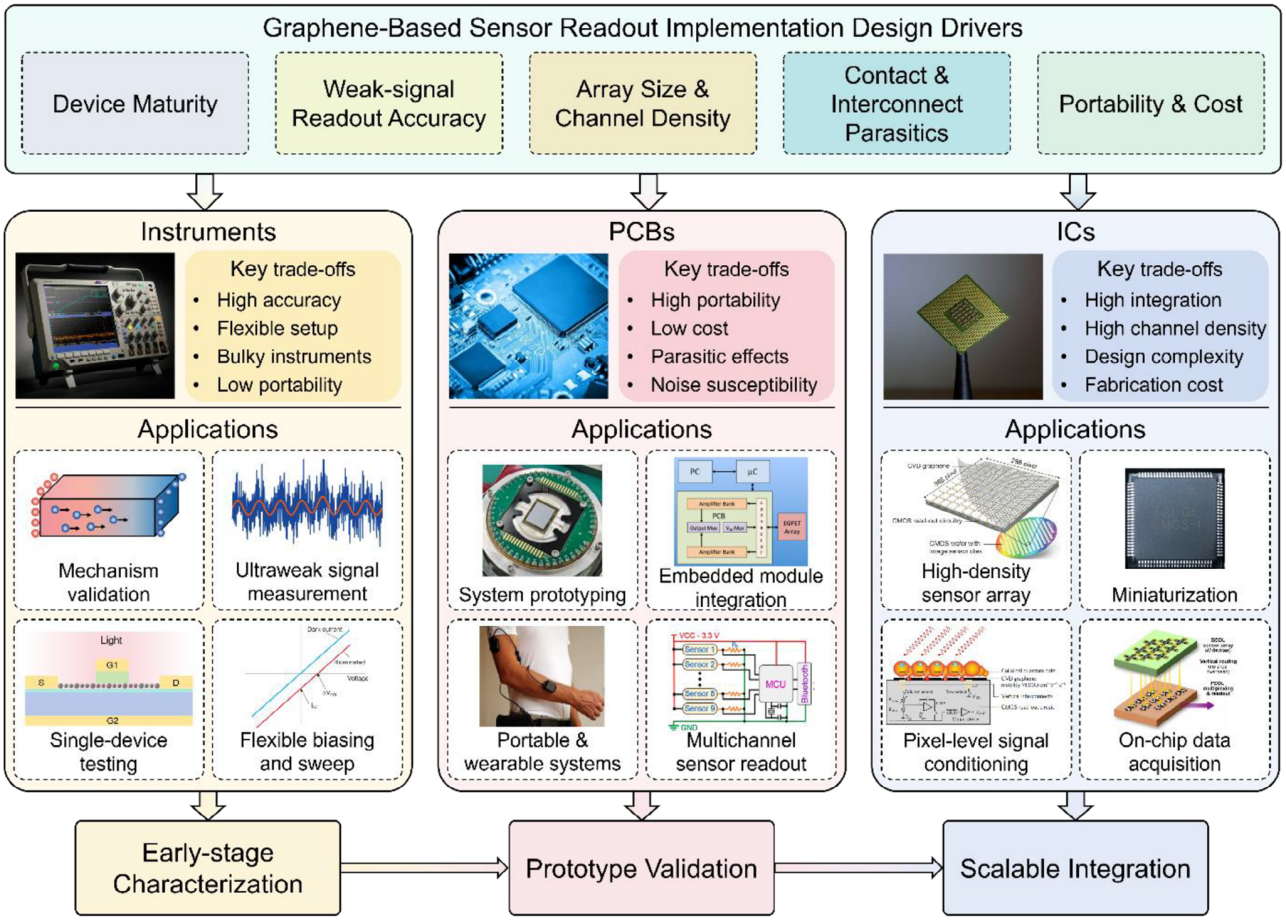
Implementation design drivers and platform selection map for graphene‐based sensor readout systems. Reproduced with permission [124]. Copyright 2024, Elsevier; Reproduced with permission [125]. Copyright 2018, American Chemical Society; Reproduced with permission [126]. Copyright 2024, IEEE; Reproduced with permission [127]. Copyright 2026, AIP Publishing; Reproduced under terms of the CC‐BY license [128]. Copyright 2026, published by John Wiley and Sons; Reproduced with permission [129]. Copyright 2017, Springer Nature.

From a system‐design perspective, graphene‐based sensor readout involves two orthogonal but coupled design axes. The first axis is determined by the implementation platforms (Figure 8), including general‐purpose instruments, printed circuit boards (PCBs) and integrated circuits (ICs). This axis determines the achievable accuracy, portability, channel density, power consumption, parasitic suppression and scalability of the complete sensing system. The second axis is determined by the electrical‐output category of the sensor (Figure 2), namely VSS, CSS, RS and CS, which mainly determines the appropriate front‐end topology. Accordingly, this section first discusses implementation platforms and graphene‐specific design drivers for readout systems, and then reviews representative front‐end topologies for VSS, CSS, RS and CS devices.

### 4.1 Implementation Platforms

The implementation platform of readout electronics is a key design variable in graphene‐based sensing systems. As summarized in Figure 7, the most relevant design drivers include device maturity, weak‐signal readout accuracy, array size and channel density, contact and interconnect parasitics, portability and cost requirements.

Instrument‐based implementations are suitable for early‐stage device characterization. At this stage, the graphene‐based sensing mechanisms, biasing conditions and output behaviors often remain under investigation. Semiconductor analyzers, source meters, impedance analyzers and oscilloscopes provide high measurement accuracy, flexible biasing and convenient parameter sweeps. However, instrument‐based platforms are bulky, expensive and difficult to use in portable, wearable or multichannel systems, which restricts their use in scalable deployment.

PCB‐based implementations are well suited to prototype validation and application‐oriented system demonstration. At this stage, the basic sensing mechanisms and output characteristics of graphene‐based sensors have usually been verified, while the readout system still requires flexible circuit configuration. PCB‐based readout is particularly attractive for portable, wearable, and cost‐effective graphene sensing systems because analog front‐end circuits, ADCs, microcontroller units (MCUs), wireless modules, and power management units can be readily integrated on PCB platforms. However, PCB platforms are still susceptible to parasitic capacitance and resistance, as well as electromagnetic interference. In addition, as the number of sensing channels increases, the required circuit modules occupy a rapidly growing board area, making PCB‐based implementations less suitable for large‐scale sensor arrays.

IC‐based implementations, typically fabricated in complementary metal oxide semiconductor (CMOS) technology, are particularly advantageous for scalable integration and miniaturized sensing systems. At this stage, the graphene‐based sensor system usually requires high channel density, low power consumption, reduced interconnect parasitics, and compact integration with signal conditioning and data acquisition circuits. CMOS‐compatible readout ICs can integrate multiple modules such as low‐noise amplifiers, transimpedance amplifiers (TIAs), multiplexers and ADCs close to the sensing elements, thereby enabling channel‐level signal conditioning. However, IC‐based platforms involve higher design complexity, fabrication cost, and development time. In addition, graphene transfer and process compatibility must be carefully considered, which remains a major challenge for practical graphene‐CMOS integration.

In this review, instrument‐based platforms are mainly considered as characterization tools for early‐stage device and mechanism validation. The following discussion therefore focuses primarily on PCB‐level and IC‐level readout methods, because these two implementation routes are more directly related to practical graphene‐based sensing systems.

### 4.2 Readout Circuit Topologies for VSS

For graphene‐based VSS, a key readout challenge is the accurate acquisition of low‐amplitude voltage variations while maintaining sufficient dynamic range and suppressing intrinsic noise and external interference. Most conditioning chains therefore combine a high‐gain amplification path with dedicated interference mitigation modules, because amplification alone can magnify noise and drift as much as the desired signal. Therefore, the selection of a VSS front end is governed not only by the required gain, but also by the sensor output amplitude, source impedance, common‐mode voltage, offset, signal bandwidth, and dominant noise spectrum. According to the principal signal conditioning strategy, existing VSS readout circuits can be broadly grouped into three representative categories: direct voltage amplification, modulation‐ and sampling‐assisted readout, and reconfigurable offset cancellation.

**FIGURE 9:**
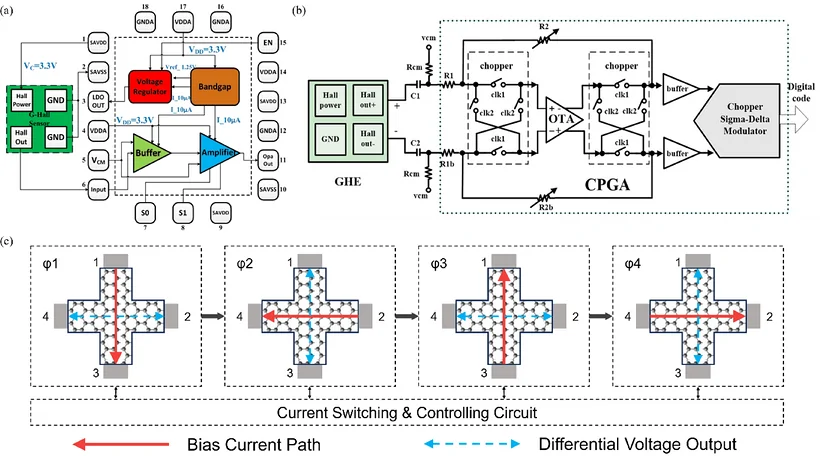
Representative readout strategies for graphene‐based VSS. (a) Direct voltage amplification: readout architecture for graphene Hall elements incorporating a buffer, a voltage amplifier, a bandgap reference and a voltage regulator. Reproduced under terms of the CC‐BY license [130]. Copyright 2014, published by Springer Nature; (b) Modulation‐ and sampling‐assisted readout: readout circuit architecture combining a CPGA and a CSDM for graphene Hall elements. CHS suppresses the circuit offset and low‐frequency noise generated in the amplification and conversion stages. Reproduced with permission [134]. Copyright 2020, American Chemical Society; (c) Reconfigurable offset cancellation: conceptual illustration of four‐phase current spinning in a graphene Hall element. The current‐injection and voltage‐sensing terminal pairs are sequentially reconfigured, and the phase outputs are combined to suppress the offset [136].

The most straightforward approach is direct voltage amplification. Operational amplifiers (OPAs), instrumentation amplifiers (INAs), and fully differential amplifiers (FDAs) are commonly employed to increase the sensor voltage to a level suitable for the ADC. OPAs provide a simple solution for single‐ended voltage acquisition, whereas INAs and FDAs are particularly suitable for differential outputs because of their high input impedance and common‐mode rejection ratio (CMRR). The amplifier design requires trade‐offs among gain, bandwidth, input‐referred noise, linearity, dynamic range, and power consumption. Programmable gain may also be required when the signal magnitude varies substantially among sensing conditions. Huang et al. [130] employed heterogeneous integration to directly combine graphene Hall elements with silicon‐based readout circuits, including voltage regulation, a bandgap reference, a buffer, and a voltage amplifier (Figure 9a). The weak Hall voltage is buffered and subsequently amplified with selectable gain. Direct differential amplification is also widely used in graphene‐based wearable biopotential sensing. Yapici et al. [131] reported the design of an electrocardiogram (ECG) readout circuit based on graphene‐functionalized textile electrodes and an INA‐based differential front end. The amplified signal was digitized by an MCU and transmitted to a PC through a Bluetooth module, enabling offline analysis while reducing the number of analog filtering components and thus lowering both circuit size and power consumption. Similarly, Golparvar et al. [132] developed a two‐stage amplification circuit for electrooculography (EOG) signal acquisition, incorporating analog high‐pass and low‐pass filters to suppress resting potential, baseline drift, and high‐frequency noise, followed by MCU acquisition and digital smoothing.

The second approach, modulation‐ and sampling‐assisted readout, employs modulation or sampling to separate the desired signal from noise, offset, and baseline drift. Lock‐in amplification (LIA) is effective when the stimulus or the sensor operating point can be periodically modulated, because phase‐sensitive detection (PSD) extracts the response within a narrow frequency band around a known reference frequency. Integrating the voltage over a prescribed acquisition interval can reduce broadband noise, although a longer integration time correspondingly limits the measurement bandwidth. Auto‐zeroing (AZ) periodically estimates and subtracts amplifier offset and low‐frequency drift, chopper stabilization (CHS) modulates the signal before amplification and demodulates it after amplification to reduce amplifier offset and 1/*f* noise, and correlated double sampling (CDS) subtracts two temporally separated samples to suppress correlated offset or baseline components [133]. These methods improve low‐frequency stability and SNR, but introduce additional circuit complexity and bandwidth limitations. Dai et al. [134] reported a representative graphene‐specific implementation of this strategy by incorporating a chopper‐stabilized programmable gain amplifier (CPGA) together with a 14‐bit chopper‐stabilized second‐order sigma‐delta modulator (CSDM), as illustrated in Figure 9b. The differential Hall voltage is first modulated and amplified by the CPGA and is subsequently converted into a digital code by the CSDM. In chopper stabilization, the low‐frequency input signal is periodically modulated before amplification and synchronously demodulated afterward, thereby restoring the desired signal to the baseband while shifting amplifier offset and low‐frequency 1/*f* noise to the chopping frequency for subsequent filtering. Meanwhile, the programmable gain determined by the feedback resistors R2 and R2b accommodates variations in the Hall‐voltage magnitude. Chopping in the CSDM further suppresses low‐frequency errors generated within the conversion stage. Another approach was demonstrated by Uzlu et al. [135] using an AC‐modulated top‐gate voltage and LIA. The gate voltage periodically drives graphene across the CNP between the p‐type and n‐type regimes, causing the Hall voltage to alternate between sensitivity extrema of opposite polarity. Synchronous demodulation therefore extracts the difference between the two states, allowing both sensitivity maxima to contribute and approximately doubling the effective Hall sensitivity. At the same time, the static magnetic‐field response is translated to the gate‐modulation frequency, where the influence of low‐frequency 1/f noise is lower than under quasi‐static measurement.

The third approach, reconfigurable offset cancellation, suppresses baseline error by periodically reconfiguring the sensor excitation and measurement terminals. This strategy is particularly relevant to graphene Hall sensors, in which geometrical asymmetry, contact mismatch, and material nonuniformity may produce a static offset that cannot be eliminated solely by increasing amplifier performance. Izci et al. [136] developed a programmable spinning‐current technique, in which the current‐injection and voltage‐sensing directions of the graphene Hall element are periodically interchanged, as illustrated in Figure 9c. Specifically, current‐switching and voltage‐switching circuits sequentially reconfigure the current‐injection and voltage‐sensing terminal pairs of the four‐terminal Hall element. The voltage outputs obtained in different phases are then combined so that the magnetic‐field‐dependent Hall component is retained, whereas a large proportion of the static sensor offset is canceled. The spinning‐current technique is therefore complementary to CHS; the former primarily suppresses offset originating within the Hall element, whereas the latter mainly reduces offset and low‐frequency noise introduced by the electronic front end.

### 4.3 Readout Circuit Topologies for CSS

Current‐source‐type sensors convert the target physical quantities into current signals, whereas the subsequent signal processing stages typically operate on voltage inputs. Readout circuits for CSS therefore share a first‐order requirement: most front ends include a current‐to‐voltage (*I*–*V*) conversion stage, and the circuit then conditions the converted voltage through amplification, filtering, and noise‐mitigation modules that match the signal bandwidth and noise environment of the specific application.

Among available *I‐*‐*V* conversion approaches, resistive and capacitive methods are the most widely adopted [137]. The resistive method is based on Ohm's law, where the output current of CSS is passed through a precision resistor and the resulting voltage drop is measured, yielding a linear *I‐*‐*V* conversion. This approach offers a practical advantage because it largely preserves the sensor's original frequency content, which makes it attractive when temporal fidelity matters. However, its performance is constrained by the amplitude of the input current because very weak currents can fall below the noise floor, and it is generally more suitable for relatively strong current signals. Readout circuits employing the resistive method are generally categorized into two main architectures: direct resistance conversion and resistive transimpedance amplifier (RTIA) configurations [138]. The direct resistance conversion approach offers simplicity and ease of implementation, but suffers from low conversion efficiency and a limited input signal amplitude range. The RTIA architecture, by contrast, introduces a parallel feedback mechanism that significantly reduces input impedance, thereby improving current injection efficiency. This improvement, however, comes at the cost of higher power consumption, making RTIA more suitable for applications that prioritize performance over energy efficiency.

**FIGURE 10:**
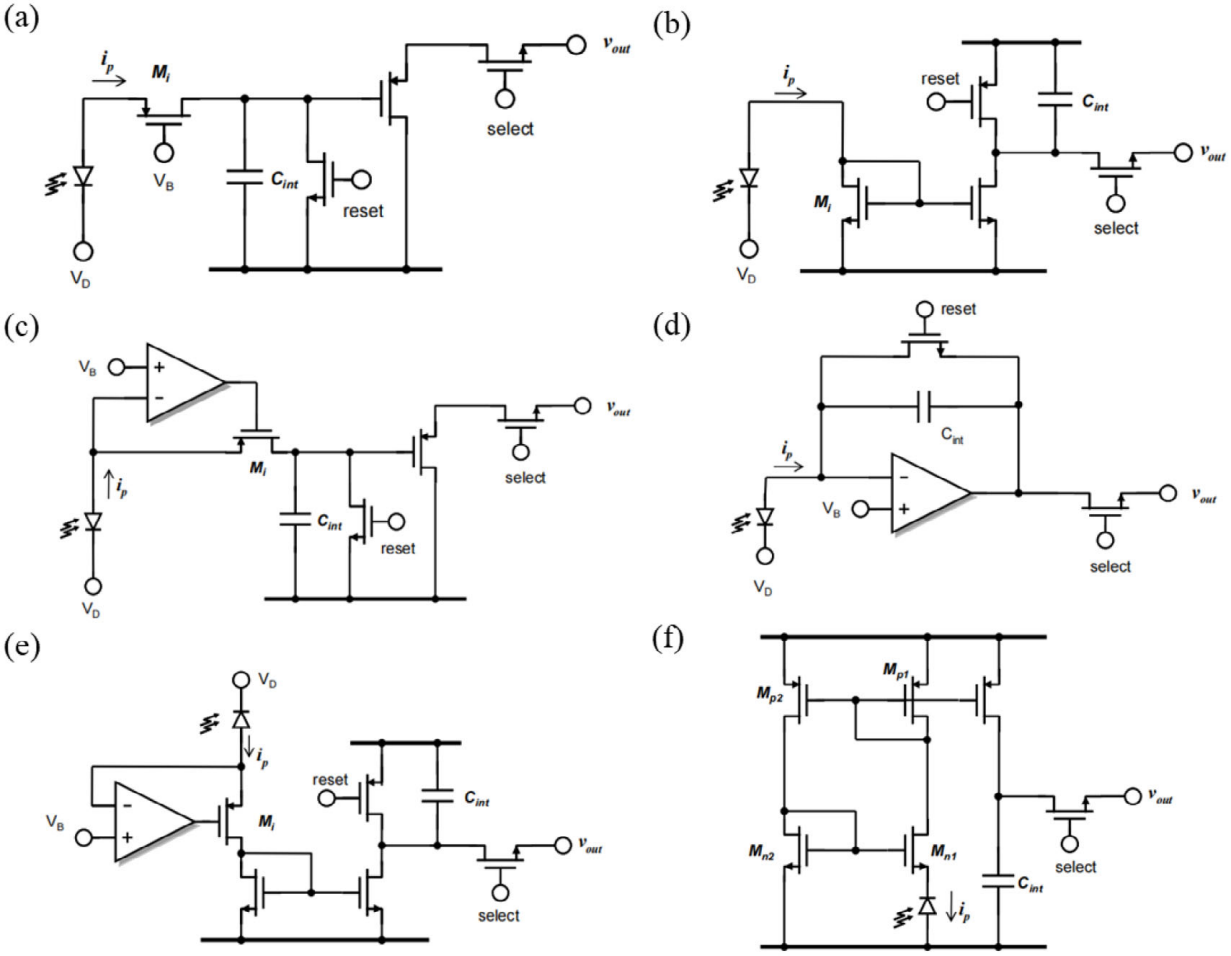
Representative circuit architectures for *I*–*V* conversion using the capacitive method. Reproduced with permission [140]. Copyright 2009, SPIE. (a) Direct injection (DI); (b) Gate modulation input (GMI); (c) Buffered direct injection (BDI); (d) Capacitive transimpedance amplifier (CTIA); (e) Buffered gate modulation input (BGMI); and (f) Current mirroring integration (CMI).

In contrast, the capacitive method operates on the principle of charge accumulation on a capacitor, where the output voltage reflects the time integral of current. This integration‐based conversion delivers excellent sensitivity for weak current signals and makes the method particularly useful in low SNR environments. The drawback, however, lies in the relatively slow response speed due to the integration process, leading to the potential loss of high‐frequency components. Designers therefore often select capacitive conversion in applications that prioritize accuracy over bandwidth or that operate at low measurement speeds. Capacitive conversion approaches [139, 140] encompass a broader range of architectures, including direct input (DI) [141], gate modulation input (GMI) [142], buffered direct input (BDI) [143], capacitive transimpedance amplifier (CTIA) [144], buffered gate modulation input (BGMI) [145], and current mirroring integration (CMI) [146], as illustrated in Figure 10. DI and CTIA are used most frequently because they represent two ends of a practical design spectrum. The DI structure is characterized by its simplicity and low power consumption, but its current injection efficiency is limited, and its bias stability is relatively poor, restricting its use to scenarios with strict requirements on circuit area and energy efficiency. By contrast, the CTIA architecture achieves lower input impedance and higher current injection efficiency through a capacitive feedback mechanism, while simultaneously providing stable bias conditions. Its disadvantages lie in higher circuit complexity and power consumption, but these trade‐offs make it particularly well suited to applications demanding high accuracy and performance.

**FIGURE 11:**
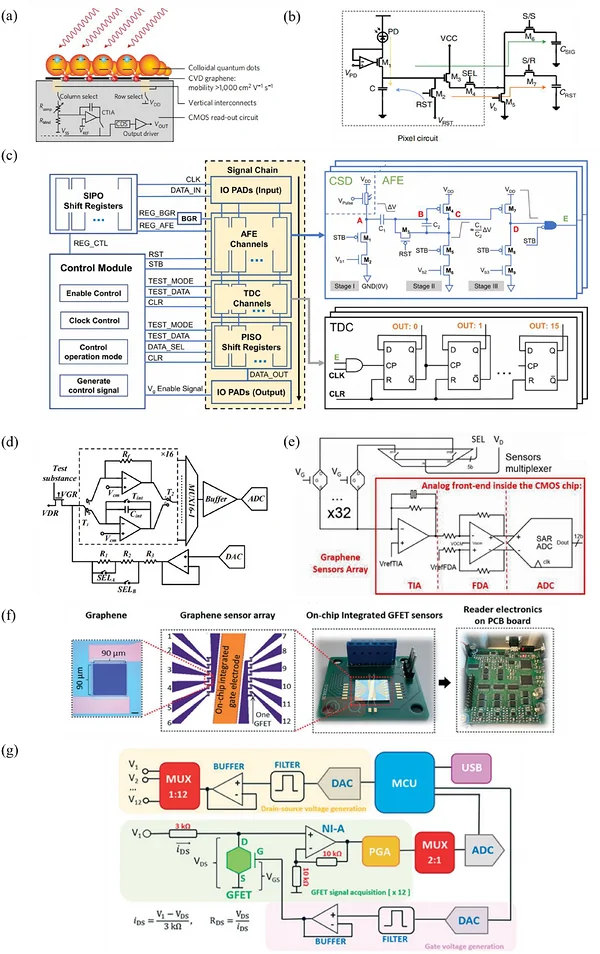
Representative readout strategies for graphene‐based CSS. (a) Capacitive method: monolithically integrated balanced readout circuit for PbS quantum dot/graphene photodetectors, employing a CTIA to integrate the differential photocurrent into an output voltage, together with blind‐pixel compensation and CDS for noise suppression. Reproduced with permission [129]. Copyright 2017, Springer Nature; (b) Capacitive method: pixel‐level readout architecture based on BDI and CDS. Reproduced with permission [148]. Copyright 2022, Springer Nature; (c) Capacitive method: digital readout circuit for graphene‐silicon charge‐sampling devices (CSDs), in which the sensor‐generated charge is sampled capacitively and amplified through a multistage analog front end, followed by TDC and PISO data transmission. Reproduced with permission [126]. Copyright 2024, IEEE; (d) Hybrid resistive and capacitive method: wide‐dynamic‐range readout architecture for a graphene EGFET sensor array, employing switchable RTIA for continuous *I*–*V* conversion and CTIA for the integration of weak currents. Reproduced with permission [149]. Copyright 2021, IEEE; (e) Resistive method: multiplexed readout circuit for graphene‐based DNA sensor array, employing an RTIA for continuous *I*–*V* conversion, an FDA for differential signal conditioning, and an ADC for digitization. Reproduced with permission [150]. Copyright 2023, IEEE; (f,g) Resistive method: physical prototype and circuit architecture for a multichannel GFET‐based biosensing system, in which current variations are converted into voltage through direct resistance conversion and conditioned by a two‐stage analog front end, while a DAC provides programmable sensor excitation and multiplexers enable flexible channel selection. Reproduced under terms of the CC‐BY license [152]. Copyright 2022, published by Royal Society of Chemistry.

In photoelectric sensing, the output‐current range and required bandwidth depend strongly on the sensing mechanism and operating conditions. Accordingly, the *I‐*‐*V* conversion architecture should be selected according to both current level and bandwidth requirements: resistive conversion is generally suitable when bandwidth is prioritized, whereas capacitive integration can improve the resolution of lower‐level currents at the expense of bandwidth. Shultz et al. [147] developed a dedicated readout circuit for lead sulfide quantum dot (PbS QD)/graphene photodetector arrays, implemented on an Arduino development board. This design employed direct resistive conversion, enabling multi‐sensitivity and multi‐channel current‐to‐voltage conversion through programmable resistors, with FDA applied for further signal conditioning. The system achieved a frame rate of 130 fps, thereby supporting relatively high‐speed imaging of detector arrays. Goossens et al. [129] introduced a balanced readout strategy for a similar sensor. As illustrated in Figure 11a, each pixel incorporates a blind pixel resistor (*R*_blind_) and an adjustable compensation resistor (*R*_comp_) in the front‐end circuit. The differential current is integrated and amplified using a CTIA, while a CDS module is applied for filtering and noise reduction. This design achieved, for the first time, monolithic integration of graphene with silicon‐based readout circuits on a single chip, offering pixel‐level digital control and effectively addressing channel uniformity issues. Liu et al. [148] extended this approach to a large‐scale array with 640 × 512 pixels. As shown in Figure 11b, the pixel‐level readout employed a BDI structure for signal integration, combined with CDS for noise suppression. By adjusting the integration time to accumulate carriers excited under varying illumination intensities, high‐quality imaging of large‐scale graphene‐based photodetector arrays was realized. Wang et al. [126] designed a readout circuit based on a graphene‐silicon charge‐sampling device (CSD), with the system architecture shown in Figure 11c. The circuit comprises an analog front‐end amplifier with three stages, a time‐to‐digital converter (TDC), and a parallel‐in‐serial‐out (PISO) shift register module. Controlled by a field‐programmable gate array (FPGA) and optimized bias voltages, this system demonstrated the ability to detect ultraweak optical signals as low as 573 pW at 808 nm, highlighting its potential for photodetection with ultrahigh sensitivity.

In molecular sensing, current signals from graphene‐based CSS are often weak and require precise separation from the baseline. At the same time, the increasing demand for portable detection platforms necessitates that readout circuits combine high performance with low power consumption, compact size, and cost efficiency. This requires careful optimization of baseline recovery modules, differential amplification stages, and power‐efficient architectures. Zhang et al. [149] reported a readout circuit based on graphene electrolyte‐gated field‐effect transistors (EGFETs) DNA sensor array, as shown in Figure 11d. The design integrates both CTIA and RTIA modules, along with a baseline elimination circuit, enabling dynamic switching across a wide input current range from 20 pA to 10 µA. This broad dynamic range allows DNA detection with extremely high throughput. Pinzon et al. [150] developed a readout circuit for a GFET‐based DNA sensor operating in DC mode, with its architecture shown in Figure 11e. The system comprises a TIA, an FDA, and an ADC, which together provide current‐to‐voltage conversion, common‐mode noise suppression, and digitization. This design supports multiplexed signal readout, making it particularly well suited for large‐scale array sensors. Laliberté et al. [151] designed a portable readout circuit for a GFET‐based interleukin‐6 (IL‐6) biomarker sensor. Employing a multistage amplifier architecture, the circuit achieves ultralow bias voltage (below 50 µV) and low noise (below −90 dBV). The front‐end stage operates at zero bias, continuously correcting the DC offset of the main amplifier. This configuration is well suited for integration into standalone wearable devices, underlining its application potential in portable biosensing. Xu et al. [152] developed a portable multichannel readout system for a GFET‐based SARS‐CoV‐2 biosensor. The system architecture, illustrated in Figure 11f,g, consists of 12 independent channels and employs a two‐stage amplification scheme for signal acquisition. A digital‐to‐analog converter (DAC) generates excitation signals with adjustable amplitude and bandwidth, while multiplexers provide flexible channel switching. This design supports multiple measurement modes, offering high precision and portability, and is thus suitable for rapid virus detection. Mackin et al. [125] designed a multichannel readout circuit for a GFET‐based ammonia gas sensor array. The circuit employs an RTIA and an OPA to convert and amplify sensor output currents into voltage signals, with data acquisition carried out via an MCU. The integration of graphene sensor arrays in an insertable chip with customized PCBs enabled convenient monitoring of large‐scale sensor arrays simultaneously, providing an efficient solution for high‐throughput gas detection.

### 4.4 Readout Circuit Topologies for RS

Resistor‐type sensors operate by converting physical quantities into measurable variations in resistance. A readout circuit cannot directly “read” resistance in the same way that it reads a voltage waveform, so the front end must first perform signal conversion. In this conversion stage, resistance changes are translated into secondary parameters such as voltage, current, time interval, or frequency. These converted signals can then be further processed through standard amplification and filtering modules. Representative RS readout architectures can be understood through three complementary strategies: biasing‐based method, bridge‐based method, and resistance‐to‐time/frequency conversion [153, 154, 155].

The most direct strategy is the biasing‐based method. Under constant‐current excitation, the sensor resistance is converted into a voltage, while under constant‐voltage excitation, the sensor resistance determines the current, which can subsequently be converted into a voltage using a resistive or capacitive method described in Section 4.3. Voltage‐divider configurations are also widely used because of their simple structure and straightforward implementation. Although simple to implement, voltage‐divider readout may become less suitable for high‐precision measurements when reference‐resistor errors, excitation variations, and limited sensitivity to small resistance changes are significant.

The bridge‐based method is based on the principle of voltage division by resistance, with the Wheatstone bridge [156] being the most widely adopted configuration. A balanced bridge converts a small resistance variation into a differential voltage, which is subsequently amplified by an INA or FDA. Compared with biasing‐based methods, the bridge‐based method can provide improved sensitivity to small relative resistance changes and suppress disturbances that similarly affect the matched bridge arms. However, its actual common‐mode and drift suppression also depends on bridge matching, excitation signal stability, and reference resistor accuracy.

The resistance‐to‐time/frequency conversion uses the sensor resistance as part of an oscillation or a timing circuit, and the readout then represents resistance variation through changes in time interval, pulse width, duty cycle, period, or oscillation frequency. Typical implementations include RC oscillators [157], ring oscillators [158], and relaxation oscillators [159]. For example, in a relaxation oscillator circuit [155], the resistor is biased to generate a current, which is then integrated on a capacitor; the voltage across the capacitor is compared to a reference, and a comparator then toggles when the capacitor voltage crosses a reference; the resulting square wave duty cycle then reflects the resistance value. In a ring oscillator, the sensing resistance changes the propagation delay of the inverter stages and consequently modifies the oscillation frequency. The resistance‐to‐time/frequency conversion facilitates direct interfacing with MCUs and FPGAs, while reducing dependence on the absolute voltage gain of the analog amplification chain. It can provide a wide dynamic range and high resolution, although the achievable performance is limited by oscillator stability, timing jitter, and circuit drift. Increased circuit complexity and implementation cost are additional drawbacks, and periodic calibration may be required to maintain high measurement accuracy during long‐term operation.

**FIGURE 12:**
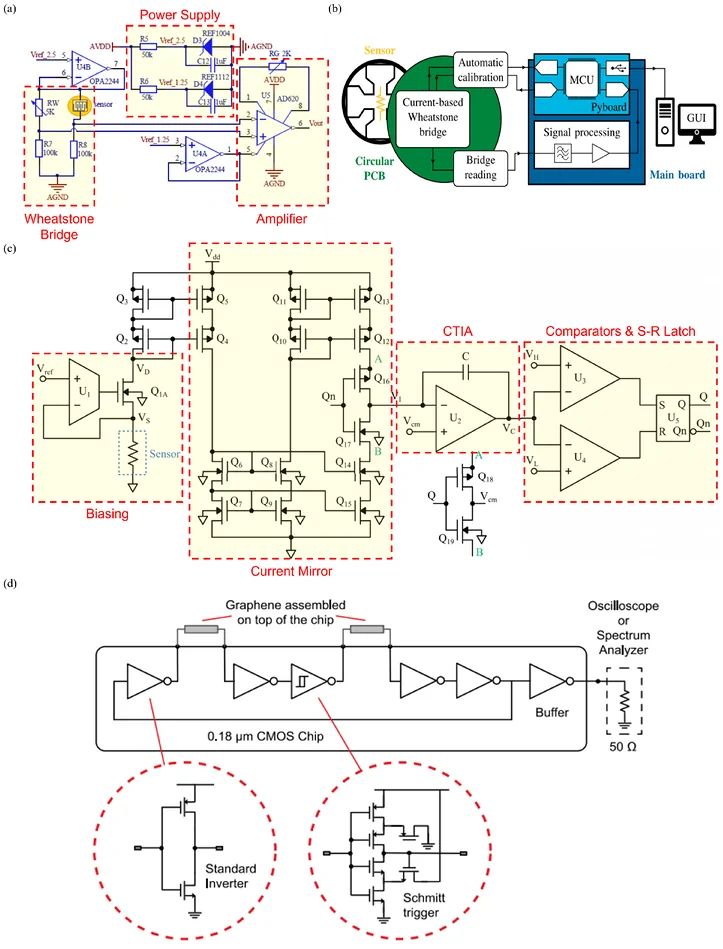
Representative readout strategies for graphene‐based RS. (a) Bridge‐based method: readout architecture for a ZnO‐rGO heterojunction ammonia sensor, in which the sensing element is incorporated into a Wheatstone bridge and the bridge output is acquired and processed by an MCU. Reproduced with permission [160]. Copyright 2015, IEEE; (b) Bridge‐based method: readout system for rGO‐based piezoresistive pressure sensors, incorporating an MCU‐controlled auto‐calibration loop to compensate for initial bridge imbalance and baseline drift, followed by amplification, filtering, and ADC. Reproduced under terms of the CC‐BY license [168]. Copyright 2024, published by IEEE. (c) Resistance‐to‐time/frequency conversion: readout architecture for chemiresistive gas‐sensor arrays, including a biasing circuit converting resistance into current, followed by a current mirror, CTIA, comparator, and SR‐latch. The resulting square‐wave period is approximately proportional to the sensor resistance. Reproduced with permission [162]. Copyright 2022, IEEE; (d) Resistance‐to‐time/frequency conversion: schematic of a monolithically integrated graphene‐CMOS gas sensor employing a five‐stage ring oscillator. Gas‐induced resistance variations modify the stage propagation delay and consequently shift the oscillation frequency. Reproduced under terms of the CC‐BY license [163]. Copyright 2017, published by Springer Nature.

These three strategies have been applied to graphene‐based molecular, mechanical, and thermal sensors. In molecular sensing, the resistance response range of graphene‐based molecular sensors varies significantly with different molecules, with the resistance changes spanning up to three orders of magnitude. Consequently, the design of readout circuits must simultaneously accommodate a wide working range and high sensitivity. One representative approach employs a Wheatstone bridge cascaded with an INA, enabling resistance‐to‐voltage conversion and extraction of weak differential signals through amplifiers with high CMRR (>100 dB). As an alternative, resistance‐to‐time/frequency conversion demonstrates advantages in terms of integration and detection accuracy. Sun et al. [160] developed a ZnO‐rGO heterojunction ammonia sensor, where the combined design of a Wheatstone bridge module, which turns the resistance variation into the differential voltage, and an amplifier with power supply circuits provided a universal platform for real‐time, ultrasensitive, and reliable gas detection at room temperature (Figure 12a). Li et al. [161] designed an MXene‐graphene COVID‐19 sensor that incorporated an auto‐balancing bridge and dual‐mode voltage divider. This design enabled dynamic adaptation to resistance changes from 10 Ω to 10 MΩ and effectively addressed the dynamic range limitations inherent to conventional single‐mode circuits. Puyol et al. [162] reported a readout circuit for graphene gas sensor arrays with low power consumption (Figure 12c). A feedback‐controlled biasing stage maintains a constant voltage across the sensor, thereby converting its resistance into a current inversely proportional to the sensor resistance. This current is scaled by cascoded current mirrors and alternately directed into a CTIA. When the CTIA output reaches the upper or lower threshold, the corresponding comparator toggles an SR latch and reverses the integration‐current direction, producing a square‐wave digital output. Consequently, the oscillation period is approximately proportional to the sensor resistance, whereas the frequency varies inversely with resistance. The circuit accommodated resistances from 1 kΩ to 33 MΩ with a maximum current consumption of 194 µA and was experimentally validated using a PANI sensor for NH_3_ detection and a pristine graphene sensor for NO_2_ detection. Zanjani et al. [163] developed a monolithic graphene‐CMOS gas sensor (Figure 12d) based on a five‐stage ring oscillator. Gas adsorption induces charge transfer in graphene and changes its resistance, thereby modifying the RC propagation delay of the oscillator stages: an increase in graphene resistance increases the propagation delay and reduces the oscillation frequency, whereas a resistance decrease produces the opposite response. A Schmitt‐trigger inverter was incorporated into the third stage to improve the internal voltage swing and reduce frequency jitter, while a final buffer drove the external measurement equipment. The integrated platform enabled frequency‐domain detection in the 2 ppm range for NO_2_, demonstrating direct resistance‐to‐frequency conversion within a graphene‐CMOS architecture.

In mechanical sensing, the sensing information is commonly encoded as changes in resistance, which may be small relative to the baseline and therefore require readout circuits with sufficient resolution and low noise. Since these devices are often applied in wearable and flexible electronics, additional requirements include structural simplicity, low power consumption, and compact design. Zhu et al. [97] employed a Wheatstone bridge to detect resistance variations in graphene‐based piezoresistive sensors, offering a straightforward readout solution. Buraioli et al. [164] developed a readout circuit for pulse wave velocity measurement using graphene pressure sensors. Their design included a biasing circuit, a TIA, and a high‐pass filter, achieving resistance‐to‐current conversion, current‐to‐voltage conversion, and noise suppression. The circuit was low‐cost, compact and well‐suited for wearable health monitoring. Feng et al. [165] designed a readout circuit for flexible graphene pressure sensors, where resistance changes were first converted into voltage signals through a biasing circuit, followed by multistage amplification and filtering for effective detection of weak pressure pulses. Li et al. [124] reported an rGO/porous silicone elastomer pressure sensor integrated with the readout architecture including a voltage divider and a Bluetooth module controlled by an MCU for wireless data transmission, enabling flexible interaction between the human and machine. Wang et al. [166] applied a Wheatstone bridge to a graphene/silicon nitride suspended membrane flow sensor, achieving the readout with a large dynamic range. Similarly, Lamba et al. [167] employed a Wheatstone bridge for graphene piezoresistive force sensors, achieving precise and stable signal acquisition. Sanginario et al. [168] developed a readout system for rGO‐based piezoresistive pressure sensors using a self‐balancing current‐mode Wheatstone bridge, together with differential signal conditioning, a programmable‐gain instrumentation amplifier, and analog filtering (Figure 12b). Before signal acquisition, an MCU‐controlled auto‐calibration module measures the current in each bridge branch through a sensing resistor and iteratively adjusts two DAC‐generated offset voltages applied to the balancing amplifiers until the residual branch currents fall below a predefined threshold. During acquisition, the pressure‐induced differential currents are converted into voltages by TIAs, while the previously applied balancing offsets are subtracted before differential amplification, bandpass filtering, and ADC. The system achieved an SNR of 23.5 dB, enabling detection of milliohm‐level resistance changes and accurate measurement of pulse transit times. Xu et al. [169] proposed a graphene‐based noninvasive intraocular pressure (IOP) sensor. The readout circuit was designed with a micro‐nano Wheatstone bridge combined with an instrumentation amplifier. The acquired data were then wirelessly transmitted to mobile applications through a Bluetooth module, resulting in a portable and biomedical monitoring system with high sensitivity.

In resistive temperature sensing, the magnitude of the temperature‐induced resistance change depends on the sensor characteristics and the operating temperature range. When this variation is small relative to readout noise and drift, sufficient resolution and low‐drift signal conditioning are required. Liu et al. [170] designed a temperature sensor using polyaniline (PANI)‐doped graphene‐polydimethylsiloxane (PDMS) composites for respiratory monitoring. To address weak resistance changes and rapid response requirements, they developed a readout circuit with low temperature drift and fast conversion rate, incorporating a proportional amplifier and an ADC, achieving high linearity. Jung [171] reported a portable digital readout system for rGO‐based temperature sensors. In this design, a voltage divider with a fixed resistor was combined with the Arduino‐based data acquisition, which enabled temperature detection in a compact format with high sensitivity and accuracy.

Beyond these element‐level conversion architectures, algorithm‐assisted calibration and large‐scale array integration represent two important future directions for graphene‐based RS readout. In recent years, substantial advances have been reported in resistive‐sensor readout through algorithmic correction and calibration methods [172, 173, 174, 175, 176]. These approaches are expected to become increasingly important for graphene‐based sensors because their responses can be strongly affected by device variability and environmental drift. Large‐scale sensor arrays introduce an additional challenge because the system must scan many resistive elements efficiently while controlling crosstalk and hardware overhead. Two‐dimensional scanning techniques for resistive sensor networks have emerged as an important research focus [177, 178, 179, 180, 181]. Representative approaches include the inserting diode method [182], inserting transistor method [183], passive integrator method [184], resistance matrix approach [185], incidence matrix approach [186], voltage feedback method [187], and zero potential method [188]. Performance optimization in these circuits generally targets three dimensions: enhancing measurement accuracy [189, 190, 191], suppressing crosstalk between array elements [192, 193, 194] and reducing hardware complexity through optimized architectures [185, 195, 196]. However, these techniques have not yet been widely demonstrated in graphene‐based resistive sensor arrays, for which reported systems more commonly employ active selection, analog multiplexing, and multichannel acquisition. Soikkeli et al. [56], for example, proposed a large‐scale array readout platform for GFET biosensors, consisting of an 8 × 8 macroarray, with each macrocell containing an 8 × 8 microarray supporting resistive analog front‐end, capacitive analog front‐end, ion‐selective FET, and electrolyte‐insulator‐semiconductor sensors. This circuit achieved for the first time wafer‐level monolithic integration of GFET biosensing arrays with CMOS readout circuits, thereby significantly enhancing system integration. Future graphene RS systems will require the co‐design of element‐level resistance conversion, array‐level circuitry, and algorithmic calibration to simultaneously improve precision, stability, and scalability.

### 4.5 Readout Circuit Topologies for CS

Capacitor‐type sensors operate by converting physical quantities into measurable variations in capacitance. Since raw capacitance changes cannot be directly processed by electronic circuits, dedicated conversion circuits are required to translate Δ*C* into voltage or current, either in the time or frequency domain. Representative CS readout architectures can be understood through three strategies: amplitude‐domain conversion, capacitance‐to‐time/frequency conversion, and capacitance‐to‐digital conversion (CDC) [197, 198].

Amplitude‐domain conversion includes capacitance‐to‐voltage (C2V) and capacitance‐to‐current (C2I) methods. Widely used C2V circuit topologies include charge‐sensitive amplifiers (CSAs), lock‐in detection (LID), and charge‐based capacitance measurement (CBCM). CSAs and switched capacitor (SC) circuits transfer the charge variation associated with the sensing capacitance to a reference capacitor and generate an output voltage proportional to the capacitance change. These circuits provide high sensitivity but may be affected by charge injection, amplifier offset, and parasitic capacitance [199]. The LID method employs AC excitation combined with synchronous demodulation, enabling extraction of weak signals under extremely poor SNR conditions. However, the demodulation and low‐pass‐filter time constants introduce a trade‐off between noise rejection, response time, and measurement bandwidth [200]. The CBCM approach measures the charge transferred during periodic charging and discharging operations, typically through a current mirror and an integrating capacitor, and can achieve sub‐femtofarad capacitance resolution [201, 202]. The C2I method converts capacitance or differential capacitance into a current under AC or switched excitation [203]. Differential C2I implementations can provide fast response and suppress common‐mode noise.

Capacitance‐to‐time/frequency conversion uses the sensing capacitance as part of a charging or oscillation circuit and represents capacitance variations through changes in pulse width, duty cycle, period, or oscillation frequency. Typical implementations include RC oscillators, relaxation oscillators, and capacitance‐controlled ring oscillators [204, 205]. Its operating principle is analogous to the resistance‐to‐time/frequency conversion described in Section 4.4, except that the sensing capacitance, rather than the sensing resistance, determines the charging or discharging dynamics or the propagation delay of the oscillator.

CDC produces a digital code without requiring a separate external ADC following an analog‐output front end. Common implementations include the Σ–Δ modulator (SDM) and successive approximation register (SAR) architectures. In an SDM‐based CDC, SC charge transfer, oversampling, and noise shaping are used to obtain high‐resolution digital outputs, making this approach suitable for weak capacitance variations and relatively low‐bandwidth applications, though at the expense of increased power consumption [206]. SAR‐based CDCs employ iterative approximation and charge redistribution to determine the sensing capacitance without oversampling [207]. They generally provide faster conversion and lower energy per conversion, while their accuracy is affected by capacitor matching, noise, parasitic capacitance, and the resolution of the internal capacitive array. Thus, SDM‐based CDCs typically favor high resolution at relatively low bandwidth, whereas SAR‐based CDCs favor conversion speed and energy efficiency.

**FIGURE 13:**
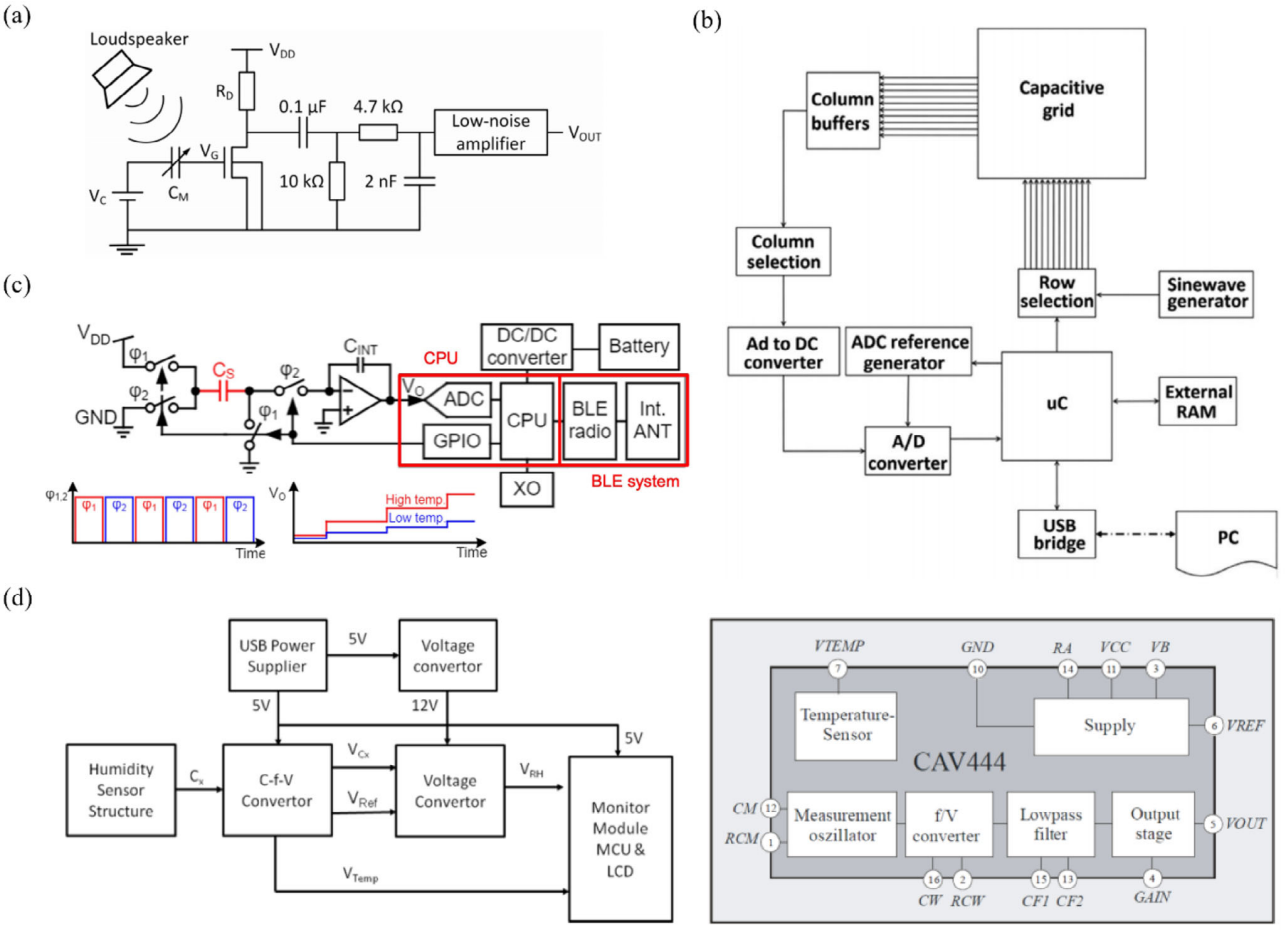
Representative readout strategies for graphene‐based CS. (a) Amplitude‐domain conversion: readout architecture for a graphene‐based MEMS microphone, in which sound‐pressure‐induced capacitance variations modulate an electrically biased sensing node and are converted into a voltage output through AC coupling, filtering, and low‐noise amplification. Reproduced with permission [209]. Copyright 2019, IEEE; (b) Amplitude‐domain conversion: AC‐excited differential readout architecture for a graphene‐based capacitive touchscreen array. Touch‐induced capacitance variations are represented by changes in the amplitude of the acquired column signals. Reproduced with permission [210]. Copyright 2021, John Wiley and Sons; (c) Amplitude‐domain conversion: readout system for graphene‐based capacitive temperature sensors, employing SC front ends together with ADC, MCU, and wireless communication. Reproduced under terms of the CC‐BY license [212]. Copyright 2022, published by AAAS; (d) Capacitance‐to‐time/frequency conversion: RC‐oscillation‐based readout architecture for a GO capacitive humidity sensor, in which humidity‐induced capacitance variations change the oscillation frequency, which is subsequently converted into an output voltage. Reproduced under the terms of the CC‐BY license [208]. Copyright 2016, published by MDPI.

These three strategies have been applied to graphene‐based capacitive sensors in molecular, mechanical, and thermal sensing applications. In molecular sensing, Feng et al. developed a prototype readout system for a GO‐based capacitive humidity sensor using an RC‐oscillation‐based frequency‐to‐voltage converter (Figure 13d) [208]. In this system, humidity‐induced capacitance variations changed the oscillation frequency of the RC interface. A CAV444 frequency‐to‐voltage converter subsequently converted the frequency variation into an output voltage, which was further processed by calibration modules. The system therefore implemented a capacitance‐to‐frequency‐to‐voltage conversion chain and provided a compact interface between the GO capacitive sensing element and conventional voltage‐domain electronics. In mechanical sensing, graphene‐based capacitive sensors are widely used in flexible‐device applications, where small capacitance changes relative to the baseline and parasitic capacitances require high‐resolution readout and careful parasitic control. As a result, their readout circuits must be both structurally simple and highly precise. Wood et al. [209] designed a readout circuit for a graphene‐based micro‐electromechanical systems (MEMS) microphone sound pressure sensor (Figure 13a). In this design, capacitance changes induced by variations in sound pressure were converted into voltage variations through a biased transistor‐based interface, followed by AC coupling, filtering, and low‐noise amplification. When operating at sound‐pressure levels of 70–80 dB, the system demonstrated a flat frequency response, highlighting its stability and reliability. Franco et al. [210] proposed a differential capacitance readout approach for graphene‐based touchscreen sensing arrays (Figure 13b). In this method, an alternating signal was applied to row electrodes and touch‐induced capacitance variations were detected from changes in the output amplitude at the column electrodes. Differential architecture combined with digital filters effectively suppresses external noise, while the parallel processing method significantly improves computational efficiency. The inclusion of gain control compensates for variability in graphene properties, and an MCU manages signal generation, data conversion, touch detection, and wireless transmission. The resulting system supports multi‐touch detection with high sensitivity, strong noise immunity and efficient processing, making it well suited for complex touchscreen applications. Shiskins et al. [94] employed a readout scheme for a graphene film array pressure sensor based on a commercial CDC, a logic level converter and an Arduino development board. Controlled by the MCU, the circuit digitized capacitance changes and achieved measurement resolution down to 10 aF against a background capacitance of several tens of pF, enabling capacitance monitoring with high precision. Kong et al. [211] developed a portable readout circuit for a droplet GO‐based pulse pressure sensor. Their approach relied on a simple RC circuit for charge and discharge, and the resulting time‐dependent signal was subsequently acquired to monitor pulse pressure. This approach offered a simple structure and convenient integration, making it suitable for portable health‐monitoring applications.

In temperature sensing, weak‐signal processing and array‐scale readout have become key directions, because practical temperature mapping requires both sensitivity and scalable integration. Kang et al. [212] reported a readout circuit for a graphene‐based temperature sensor array (Figure 13c). Implemented on a flexible printed circuit board, the system integrated front‐end readout circuits, an ADC, an MCU, multiplexers, a Bluetooth module, and power supply components. The front‐end circuit of each pixel adopted a switched‐capacitor (SC) amplifier comprising the sensing capacitor *C*_S_ and an integrating capacitor *C*_INT_, as shown in Figure 13c. During one clock phase, *C*_S_ is charged by the excitation voltage, and during the subsequent phase, the corresponding charge is transferred to *C*_INT_. A temperature‐induced change in *C*_S_ therefore changes the amount of transferred charge and produces a corresponding variation in the output voltage for subsequent digitization and processing. This system demonstrated sensitivity to capacitance changes on the order of femtofarads with response times below 0.3 s, making it highly suitable for rapid and sensitive human body temperature monitoring.

### 4.6 Emerging High‐Frequency Readout Methods

In addition to the four mainstream readout categories discussed above, researchers have developed specialized readout schemes for sensors whose outputs are encoded in high‐frequency resonant, propagation, or scattering responses rather than in quasi‐static voltage, current, resistance, or capacitance. In particular, graphene‐based sensors are increasingly being explored in acoustic, radio‐frequency, and microwave frequencies. This subsection discusses two emerging directions: acoustic‐wave‐based sensing and readout [213], and dielectric‐modulated RF/microwave sensing approaches [214].

**FIGURE 14:**
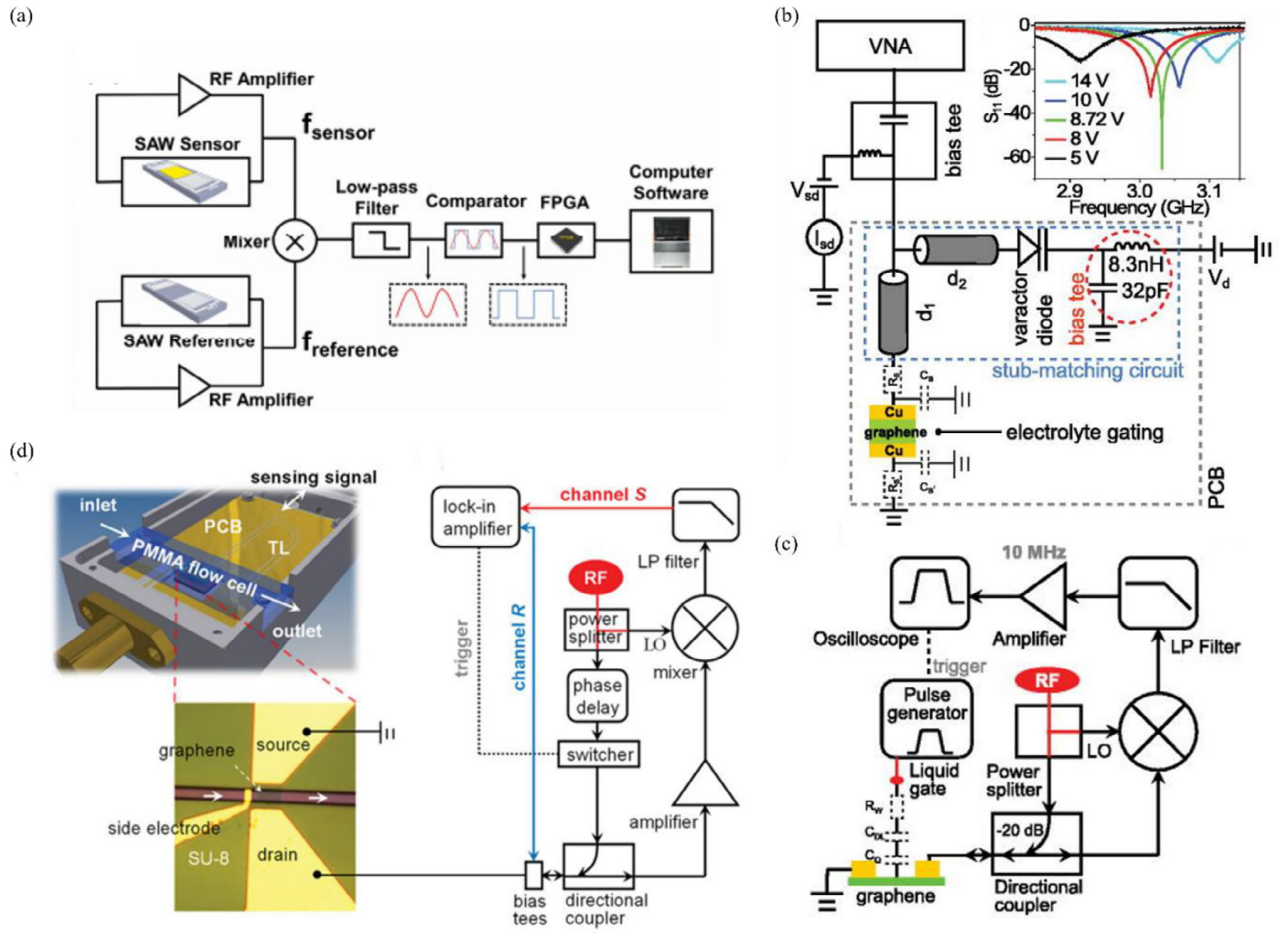
Representative emerging high‐frequency readout methods for graphene‐based sensors. (a) Differential readout for a GO‐coated SAW humidity sensor. The RF outputs of the sensing and reference SAW devices are amplified and mixed to generate a difference‐frequency signal, followed by low‐pass filtering, waveform conversion, and FPGA‐based frequency detection. Reproduced with permission [216]. Copyright 2020, IEEE; (b,c) Tunable stub‐matched RF reflectometry and homodyne time‐resolved readout for a graphene EGFET. In RF reflectometry, the d_1_‐d_2_ stub network and varactor match the device impedance to 50 Ω, while the bias tee enables simultaneous RF and DC measurements. In time‐resolved readout, a directional coupler separates the reflected RF signal, which is mixed with a coherent LO, low‐pass filtered, amplified, and recorded as a baseband time‐domain response. Reproduced with permission [218]. Copyright 2014, AIP Publishing; (d) Dual‐channel UHF readout of a graphene EGFET. The conventional resistance channel *R* and the UHF reflection‐derived channel *S* are acquired simultaneously. Reproduced with permission [219]. Copyright 2022, John Wiley and Sons.

In acoustic‐wave‐based sensors, graphene‐based materials commonly serve as stimulus‐sensitive elements, while an underlying piezoelectric structure converts perturbations of acoustic propagation into RF electrical signals in the MHz‐to‐GHz range. Molecular adsorption on graphene can affect the acoustic response through coupled mass loading, viscoelastic loading, and dielectric loading [215]. The readout system must therefore accurately extract small frequency or phase variations from a comparatively high‐frequency carrier while suppressing drift and noise. Wang et al. [216] designed a readout circuit for a GO thin‐film surface acoustic wave (SAW) humidity sensor (Figure 14a). The system employed separate sensing and reference SAW devices together with dedicated RF circuits and digital processing. The RF amplifier first enhances the sensor signal, which is then mixed with a reference signal to generate a lower difference‐frequency component. The low‐pass filter suppresses high‐frequency components of the mixed signal, retaining only the difference frequency term. The comparator digitizes this output, and the FPGA performs data acquisition and frequency detection. This differential down‐conversion architecture avoids direct digitization of the original high‐frequency SAW outputs, reduces the frequency range required by the digital backend, and also suppresses common environmental and circuit perturbations. Another architecture embeds the acoustic resonator directly into an oscillator loop. Xuan et al. developed a GO‐coated film bulk acoustic resonator (FBAR) humidity sensor together with a Pierce oscillator and an FPGA‐based counter [217]. The transistor provides loop gain, while the FBAR and feedback capacitors form the frequency‐selective network. Humidity‐induced resonance shifts are therefore converted directly into changes in the oscillator output frequency, which is measured by the FPGA. Compared with repeated sweeps, this approach enables compact and continuous frequency readout.

Dielectric‐modulated RF/microwave graphene sensors encode sensing information into frequency‐dependent electromagnetic responses. Molecular adsorption or interfacial binding can simultaneously modify the complex dielectric permittivity, dynamic conductivity, and impedance. RF reflectometry provides a high‐frequency readout route by monitoring the signal reflected from a graphene sensing device. The reflection coefficient *S*_11_ is governed primarily by the input impedance of the sensing structure relative to the characteristic impedance of the RF system, whereas the transmission coefficient *S*_21_ additionally depends on wave propagation and coupling through the structure. Because the impedance of graphene devices can be substantially higher than the conventional 50‐Ω RF environment, impedance transformation is often required to achieve sufficient sensitivity. Fu et al. demonstrated an early reflection approach for graphene EGFETs over the 2–4 GHz range using a tunable stub‐matching network [218]. As shown in Figure 14b, the electrolyte‐gate voltage modulates the device impedance, thereby changing *S*_11_ measured by the vector network analyzer (VNA). The RF signal is delivered through a bias tee, which permits simultaneous drain‐to‐source biasing and current measurement through *V*_sd_ and *I*_sd_, and then propagates along the coplanar waveguide *d*_1_ to the GFET. A shunt stub d_2_, terminated by a varactor diode, transforms the high GFET impedance toward the 50‐Ω system impedance. The on‐board 8.3‐nH inductor and 32‐pF capacitor form a bias tee that supplies the bias voltage *V*_d_ while isolating the RF and DC paths. For time‐resolved readout (Figure 14c), a pulse generator applies a 2‐MHz square‐wave perturbation to the liquid gate. A power splitter divides the 3.55 GHz carrier into an interrogation branch and a coherent local oscillator (LO) branch. The interrogation signal reaches the matched GFET through a −20 dB directional coupler, which separates the reflected wave and routes it to the RF port of the mixer. Mixing the reflected signal with the coherent LO converts the impedance‐dependent reflection variation to a near‐DC baseband voltage. The low‐pass filter removes the high‐frequency mixing product, the 10‐MHz‐bandwidth amplifier increases the signal amplitude, and the oscilloscope records the resulting time‐domain response. This architecture demonstrates that impedance matching and homodyne down‐conversion can translate small, fast impedance variations of a graphene EGFET into a readily measurable baseband signal, and also enables time‐resolved high‐frequency sensing. Building on this approach, Zhang et al. configured a graphene EGFET for simultaneous conventional resistance and ultrahigh frequency (UHF) reflectometry measurements near 2 GHz, as shown in Figure 14d [219]. The graphene channel was exposed to the analyte through a PMMA flow cell, while the chip was wire‐bonded to a PCB transmission line and enclosed in an RF box. Bias tees separated the conventional resistance signal *R* from the UHF interrogation path. For channel *S*, the RF source was divided into an interrogation signal and a coherent LO. A directional coupler delivered the carrier to the EGFET and separated the reflected wave, which was amplified, mixed with the LO, low‐pass filtered, and detected by a LIA. Since carrier‐density variations affect both *R* and *S*, with additional contributions to *S* introduced by local dielectric changes, comparison of the two channels enables the dielectric‐modulated response to be distinguished from the conventional conductance response. Gubeljak et al. integrated an electrochemically gated graphene channel into a coplanar waveguide coupled to a microfluidic channel. A VNA launched the microwave signal and measured the *S*_11_ and *S*_21_ responses from 50 MHz to 50 GHz, while analyte interactions modified wave propagation through both fringing‐field dielectric loading and gate‐dependent changes in graphene dynamic conductivity [220]. Huang et al. coated a printed graphene radio‐frequency identification (RFID) antenna with GO, whose humidity‐dependent permittivity changed the antenna impedance. An RFID reader detected the resulting variation in the phase of the signal backscattered by the impedance‐switching RFID chip [214]. Yasir et al. coupled a microstrip transmission line to a split square‐ring resonator containing a functionalized graphene film across its gap. The glucose binding changed the film impedance and shifted the resonant feature in transmission coefficient *S*_21_ [221].

Overall, emerging high‐frequency readout methods extend graphene sensing from quasi‐static measurements to frequency‐based features. Dedicated RF amplifiers, oscillators, mixers, filters, and frequency counters have enabled compact readout for acoustic‐wave sensors, whereas most dielectric‐modulated RF/microwave systems still rely on VNAs, external RF instruments, or commercial readers. Future development may focus on compact RF front ends that integrate signal generation, reflection or transmission detection, and a calibration module, while jointly optimizing noise, bandwidth, drift, power consumption, and packaging.

## 5 System‐Level Co‐Design Guidelines and Best Practices

Building on the sensing mechanisms and readout architectures discussed above, this section translates the PS–EQ–RF framework into practical system‐level design guidance. It presents an iterative co‐design workflow, maps key performance metrics onto the PS‐to‐EQ and EQ‐to‐RF stages, and further discusses readout architecture selection, low‐noise design, and mitigation of major nonidealities affecting graphene‐based sensing systems.

### 5.1 Co‐Design Workflow

**FIGURE 15:**
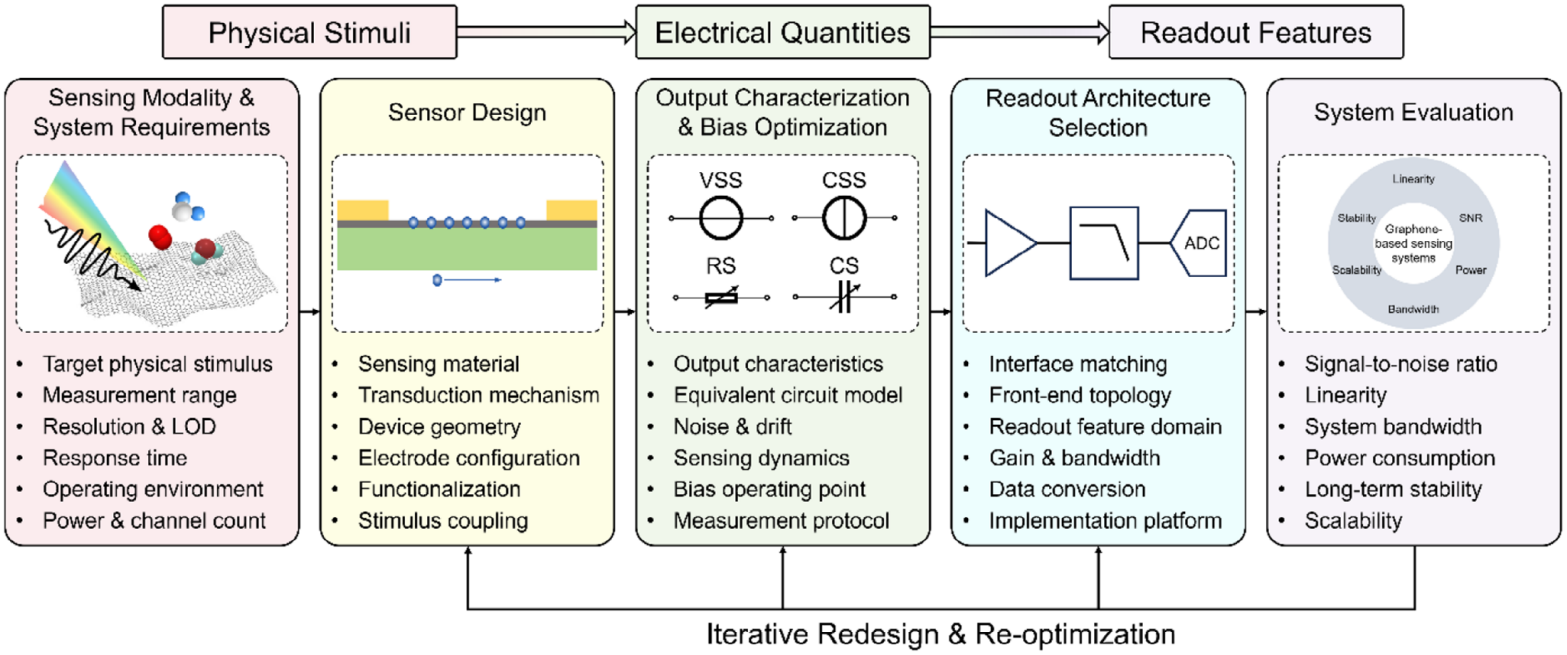
System‐level co‐design workflow for graphene‐based sensing systems within the PS–EQ–RF framework.

Graphene‐based sensing systems require a requirement‐driven and iterative co‐design process rather than a sequential approach in which the sensor is optimized independently and the readout circuit is subsequently selected. As illustrated in Figure 15, the proposed workflow comprises five stages within the PS–EQ–RF framework: definition of the sensing modality and system requirements, sensor design, output characterization and bias optimization, readout architecture selection, and system evaluation. The evaluation results are subsequently fed back to the relevant design stages for iterative redesign and re‐optimization.

The first stage is to define the sensing modality and system requirements. The target PS and intended application scenario should first be identified, followed by specification of the required measurement range, resolution and LOD, response time, and operating environment. The allowable power consumption and required channel count should also be established at this stage because they directly constrain the subsequent sensor, circuit, and implementation choices. These requirements provide the system‐level acceptance criteria against which the completed sensing system is evaluated. The second stage is sensor design, which establishes the PS‐to‐EQ conversion. An appropriate graphene‐based sensing material, such as graphene, GO, rGO, or functionalized graphene, should be selected according to the target stimulus and operating environment. The transduction mechanism, device geometry, electrode configuration, surface functionalization, and stimulus‐coupling structure should then be designed to produce a sufficiently strong and reproducible response with electrical characteristics compatible with downstream readout requirements. At this stage, the emphasis is placed on how the target PS is coupled to the sensing element and converted into a native EQ that can be effectively interfaced with the subsequent readout stage. The third stage is output characterization and bias optimization. The native output should first be identified as a voltage, current, resistance, or capacitance variation. The corresponding equivalent circuit model including VSS, CSS, RS, and CS should then be established, and the baseline level, signal range, noise characteristics, and sensing dynamics should be characterized under representative operating conditions. On this basis, the bias operating point and measurement protocol should be optimized. Relevant parameters may include the gate voltage, drain‐to‐source voltage, modulation condition, and integration time. The selected operating point should balance measurable response, noise, linearity, power consumption, and stability rather than simply maximizing the absolute sensor output. The fourth stage is readout architecture selection, which implements the EQ‐to‐RF conversion. The electrical interface should first be matched to an appropriate front‐end topology. The RF domain should then be selected according to the signal characteristics and dominant interference, with the sensing information represented through amplitude‐, time‐, or frequency‐domain features. The required gain, bandwidth, filtering, and data conversion strategy should also be determined according to the signal range and system requirements. Finally, an appropriate implementation platform, including instrument‐, PCB‐, or IC‐based implementation, should be selected by considering measurement flexibility, parasitic effects, portability, power consumption, and integration requirements. The fifth stage is system evaluation. The completed sensor‐readout system should be assessed using end‐to‐end performance metrics. The principal evaluation quantities include SNR, linearity, system bandwidth, power consumption, long‐term stability, and scalability. If the designed system does not satisfy the predefined requirements, the results should be fed back to the relevant design stages. For example, insufficient signal magnitude may require modification of the sensing material, stimulus‐coupling structure, and front‐end circuit topology. The feedback paths in Figure 15 therefore represent iterative sensor redesign, bias re‐optimization, and readout reconfiguration until the required system‐level performance is achieved.

**FIGURE 16:**
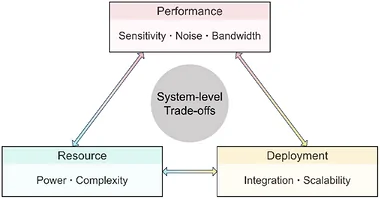
Interdependent trade‐offs governing readout architecture and system co‐design.

In addition to following the iterative workflow, readout architecture selection requires balancing several interdependent system‐level considerations, as summarized in Figure 16. These considerations can be organized into three dimensions. The performance dimension includes sensitivity, noise, and bandwidth, which collectively determine whether the system can resolve the target stimulus within the required response time. The resource dimension includes power consumption and complexity, encompassing the electrical energy and costs required by the readout system. The deployment dimension includes system integration and scalability, which determine whether the sensing system can be miniaturized, packaged, and extended to multiple channels. The relationships among these dimensions are not universal pairwise dependencies. Instead, different design choices improve selected metrics while imposing costs in other dimensions. The following discussion examines four representative groups of trade‐offs: sensitivity‐noise‐bandwidth, noise‐power‐complexity, scalability‐bandwidth‐complexity, and integration‐complexity‐flexibility. The first trade‐off exists among sensitivity, noise, and bandwidth. The usable sensitivity of a sensing system is determined not only by the magnitude of the response but also by the total noise within the measurement bandwidth. Narrowing the bandwidth or increasing the integration time can reduce integrated random noise. However, these measures reduce the bandwidth and may attenuate rapid transients. Conversely, a wider bandwidth preserves faster sensing dynamics but admits more broadband noise and generally places more demanding requirements on the front‐end amplifier and data converter. The second trade‐off involves noise, power consumption, and circuit complexity. Lower electronic noise can be achieved through increased analog bias current, low‐noise amplification, differential architectures, reference channels, correlated sampling, and modulation‐based methods. These approaches may improve the detectability of weak sensor outputs, but they require additional power and circuit area. Modulation‐based techniques can move the desired signal away from DC offsets and low‐frequency noise generated by the readout electronics. However, their effectiveness is limited by the sensor response speed and circuit complexity. The third trade‐off links scalability, bandwidth, and complexity. Multiplexing and shared front‐end or ADC resources can reduce the hardware required per sensing element and facilitate extension to large sensor arrays. Nevertheless, increasing the multiplexing ratio reduces the acquisition time and update rate available to each channel. Maintaining high per‐channel bandwidth in a large array consequently requires more parallel readout paths, or more complex scheduling and buffering. Finally, system integration is coupled to implementation complexity and design flexibility. Instrument‐based systems provide flexible excitation, broad measurement capability, and convenient modification, but their size, cost, interconnection length, and channel scalability restrict practical deployment. PCB‐based implementations provide a compromise between flexibility and portability, although board‐level parasitics, electromagnetic interference, and connector complexity become increasingly significant at low signal levels or high channel counts. IC‐based implementations can reduce interconnect parasitics, form factor, and hardware per channel while enabling dense multiplexing and local signal conditioning. These advantages are accompanied by higher design and fabrication effort, more stringent area and power constraints, reduced post‐fabrication reconfigurability, and additional challenges in graphene‐CMOS integration. These representative trade‐offs demonstrate that no single architecture simultaneously provides maximum sensitivity, minimum noise, maximum bandwidth, minimum power, minimum complexity, and unrestricted scalability and integration. The appropriate design depends on the relative priorities imposed by the target application. Importantly, co‐design does not merely select a compromise among fixed performance limits. Coordinated optimization of the sensing material, device geometry, operating point, readout bandwidth, and implementation platform can relax individual constraints and shift the achievable system‐performance frontier.

### 5.2 Mapping Metrics Onto the PS–EQ–RF Framework

The performance of a graphene‐based sensing system cannot generally be attributed exclusively to either the sensing device or the readout electronics. Instead, system‐level metrics emerge from the complete PS‐to‐EQ‐to‐RF conversion chain. Table 2 maps the principal system‐level metrics onto PS‐to‐EQ and EQ‐to‐RF stages and translates the mapping into implications for readout‐architecture selection. For each metric, the sensor‐side determinants describe the physical transduction, device, interface, and environmental factors associated with the PS‐to‐EQ conversion. The readout‐side determinants describe the loading, circuit, digitization, and system effects introduced during the EQ‐to‐RF conversion. The final column identifies representative front‐end architectures and supporting design strategies that can address the corresponding limitations. Table 2 can guide architecture selection by identifying whether the dominant limitation arises from the PS‐to‐EQ or EQ‐to‐RF stage. For example, if the noise floor is mainly determined by the amplifier, bias source, or ADC, a low‐noise front end, stable biasing, and appropriate bandwidth control are effective. However, if intrinsic noise of the sensor dominates, further reduction of circuit noise alone provides limited benefit, and differential measurement or device modification may be required.

**TABLE 2:** Mapping of key system‐level performance metrics onto the PS–EQ–RF framework and their implications for readout architecture selection.

Performance metric	Sensor‐side determinants (PS‐to‐EQ)	Readout‐side determinants (EQ‐to‐RF)	Readout architecture implications and design strategies
Noise floor	Thermal noise: channel resistance, graphene–metal contact resistance; 1/*f* noise: associated with carrier number and mobility fluctuations, trapping‐detrapping process and adsorption‐related fluctuations. External factors: operating bias point selection, self‐heating, environmental disturbances.	Circuit noise: bias‐source noise, amplifier input voltage and current noise, resistor thermal noise, *kT*/*C* noise. Digitization effects: ADC quantization and reference noise, aliasing. External factors: interchannel crosstalk and external electromagnetic interference.	Low‐noise front‐end amplifier; Stable low‐noise bias; differential readout; bandwidth limitation or integration; modulation‐ or chopping‐based detection; careful shielding and grounding.
SNR	Signal‐side: stimulus‐sensor coupling efficiency, transduction sensitivity. Noise‐side: thermal noise, 1/*f* noise within the measurement bandwidth, external factors.	Signal transfer: sensor loading, input impedance, front‐end conversion gain. Noise and digitization: circuit noise within the measurement bandwidth, digitization effects, external factors.	Front end with appropriate input impedance; gain allocation; differential readout; bandwidth limitation or integration; modulation‐ or chopping‐based detection; careful shielding and grounding.
Dynamic range	Signal span: baseline level, minimum resolvable response, maximum nonsaturating response. Range limits: saturation, nonlinearity, hysteresis, baseline drift, and device‐to‐device variability.	Analog range: front‐end input range, output swing, offset, supply voltage headroom, gain range. Digitization: ADC full‐scale range, resolution, ENOB.	Differential readout; baseline cancellation; programmable gain, auto‐ranging readout; feedback‐based operating point stabilization; sufficient input/output and supply headroom; matching of the ADC full‐scale range to the conditioned signal span.
Bandwidth	Intrinsic transduction dynamics: carrier transport, adsorption–desorption process, thermal diffusion. Device‐level limitations: output impedance, device capacitance and parasitics.	Analog path: sensor‐to‐input RC time constant, closed‐loop amplifier bandwidth, feedback parameters, analog filtering, parasitics. Acquisition path: integration time, ADC sampling rate, multiplexing ratio, digital filtering.	Wideband front‐end amplifiers; minimized sensor‐to‐input RC and parasitic delays; optimized filtering and integration time; ADC sampling rate and multiplexer settling sufficient for the required per‐channel bandwidth; Parallel readout.
Power consumption	Operating bias point; required excitation; sensor impedance; duty cycle; the number of simultaneously active sensing elements.	Supply voltage and quiescent current; bias and reference generators; ADC power; clock or oscillator power; digital processing and communication power.	Low‐voltage or current bias and excitation; self‐powered operation; event‐driven acquisition; resource sharing or multiplexed readout; power‐scalable ADC and digital processing; local computation.
Long‐term stability	Material‐side: material aging, irreversible trapping, electrode degradation; environment‐side: contamination, adsorption, encapsulation degradation.	Bias source drift; amplifier offset and gain drift; ADC reference drift; oscillator frequency drift; temperature‐induced drift.	differential readout; reference channel readout; baseline tracking; periodic calibration; feedback‐based operating‐point stabilization; environmental monitoring and compensation; appropriate thermal management.
Scalability	Manufacturability: wafer‐scale material uniformity, fabrication yield, reproducibility; array compatibility: device‐to‐device variation, sensor size, packaging compatibility.	Multiplexing ratio; Interchannel crosstalk; interconnect parasitics; ADC throughput; total power budget; data communication.	Hierarchical multiplexing or hybrid parallel readout; shared reference and ADC resources; column‐level signal conditioning; background calibration; local computation; careful channel isolation.

### 5.3 Selection Guidelines for Readout Architectures

Readout architecture selection can be organized into three successive steps. First, the front end should be matched to the sensor's native EQ. Second, the specific readout method should be selected according to the dominant system‐level constraints mapped in Table 2. Table 3 integrates these two considerations and summarizes practical guidelines for selecting representative architectures. Third, auxiliary functions, such as programmable gain, baseline cancellation, and multiplexing, should be added where necessary to address limitations not resolved by the primary conversion stage. The following discussion examines the principal performance trade‐offs among representative readout circuits for each native EQ category.

**TABLE 3:** Selection guidelines for representative readout architectures for graphene‐based sensors.

Category	Readout method	Representative architecture	Advantage	Limitation	Typical applications
VSS	Direct voltage amplification	OPA	Simple implementation; flexible gain.	Limited common‐mode rejection in single‐ended readout; amplification of offset and input noise.	Magnetic sensing [130]
		INA	High input impedance; high CMRR.	Higher complexity; gain‐bandwidth trade‐off.	Biopotential sensing [131]
		FDA	Differential signal conditioning; direct ADC interface.	Higher complexity; requires common‐mode control.	Molecular sensing [150]
	Modulation‐ and sampling‐assisted readout	LIA	Strong noise rejection; high sensitivity.	Requires modulation; narrow bandwidth.	Magnetic sensing [135]
		CHS	Suppresses electronic offset and 1/*f* noise.	Chopping ripple; clock feedthrough.	Magnetic sensing [134]
		CDS	Suppresses correlated offset and low‐frequency baseline components.	Sampling noise; reduced effective bandwidth.	Photoelectric sensing [129]
	Reconfigurable offset cancellation	Spinning‐current technique	Suppresses sensor‐originated Hall offset.	Requires multiphase switching.	Magnetic sensing [136]
CSS	Resistive method	Direct resistance conversion	Simple implementation; continuous *I*–*V* conversion.	Low conversion efficiency; limited input‐current range.	Photoelectric sensing [147] and molecular sensing [152]
		RTIA	Linear continuous *I*–*V* conversion; low input impedance.	Feedback‐resistor noise; gain‐bandwidth trade‐off.	Molecular sensing [125, 150]
	Capacitive method	DI	Small area; low power.	Low injection efficiency; poor bias stability.	—
		CTIA	High sensitivity; stable sensor bias.	Reset and *kT*/*C* noise; finite integration range.	Photoelectric sensing [129] and molecular sensing [149]
RS	Biasing‐based method	Voltage‐divider configuration	Simple structure; low cost.	Nonlinear transfer; reference‐resistor dependence.	Mechanical sensing [124] and thermal sensing [171]
	Bridge‐based method	wheatstone bridge	high sensitivity; common‐mode interference rejection.	Requires matching; continuous power dissipation.	Molecular sensing [160] and mechanical sensing [166, 167, 168, 169]
	Resistance‐to‐time/frequency conversion	Relaxation oscillator	Wide resistance range; direct frequency output.	Timing jitter and drift; limited conversion speed.	Molecular sensing [162]
		Ring oscillator	Compact CMOS integration; direct frequency output.	PVT sensitivity; oscillator jitter.	Molecular sensing [163]
CS	Amplitude‐domain conversion	AC excitation	Direct dynamic capacitance readout.	Sensitive to parasitics and noise.	Mechanical sensing [210]
	Amplitude‐domain conversion	SC	Array‐compatible; high integration compatibility.	*kT*/*C* noise; charge injection; clock feedthrough.	Thermal sensing [212]
	Capacitance‐to‐time/frequency conversion	RC oscillator	Simple frequency‐domain output.	Parasitic sensitivity; frequency drift.	Molecular sensing [208]
	CDC	Commercial CDC IC	Direct digital output; high capacitance resolution.	Sensitive to parasitic capacitance; limited input range.	Mechanical sensing [94]

For VSS, the readout strategy is mainly determined by whether the dominant requirement is basic signal amplification, suppression of electronic noise, or cancellation of sensor‐originated offset. Direct voltage amplification is the natural choice when the generated voltage can be resolved without additional modulation: OPA‐based circuits emphasize simplicity, whereas INA‐ and FDA‐based implementations provide differential conditioning when common‐mode interference or differential ADC interfacing is important. Modulation‐ and sampling‐assisted readout is introduced when direct amplification is limited by noise. LIA provides narrowband extraction of modulated signals, CHS reduces amplifier offset and electronic 1/*f* noise, and CDS suppresses correlated components in sampled signal chains. These functions may also be incorporated as auxiliary stages after another primary front end. By contrast, reconfigurable offset cancellation using the spinning‐current technique addresses offset generated within the Hall element itself. VSS readout therefore involves a trade‐off between circuit simplicity and bandwidth, and improved offset and noise suppression.

For CSS, architecture selection is governed primarily by the current magnitude and whether continuous tracking or temporal integration is required. The resistive method preserves the current waveform and is therefore suitable when temporal fidelity is important and the signal is sufficiently above the noise floor. Direct resistance conversion provides a simple implementation, whereas an RTIA offers more controlled *I*–*V* conversion but introduces feedback‐resistor noise and a gain‐bandwidth trade‐off. The capacitive method accumulates charge over time and is consequently more advantageous for weak currents. DI minimizes pixel area and power but provides limited current‐injection efficiency and bias control, whereas CTIA maintains a more stable sensor‐node potential and improves weak‐current resolution at the expense of reset‐related noise, finite integration capacity, and reduced update rate. The central CSS trade‐off is therefore bandwidth versus sensitivity.

For RS, the appropriate method depends on the relative resistance change and the preferred readout domain. Biasing‐based conversion is the simplest option and is suitable when the resistance variation is sufficiently large, although voltage‐divider implementations provide limited linearity and depend on the reference resistance. Bridge‐based conversion is more appropriate for small changes around a large nominal resistance because it converts the imbalance into a differential voltage and facilitates reference‐assisted suppression of common disturbances. Its accuracy, however, depends on bridge balance and element matching. Resistance‐to‐time/frequency conversion replaces precision amplitude measurement with period or frequency measurement, thereby facilitating digital interfacing and CMOS integration. RS selection therefore reflects a trade‐off among circuit simplicity, resolution, and digital integration.

For CS, the architecture is selected according to whether capacitance information is most effectively represented in the amplitude, time/frequency, or digital domain. Amplitude‐domain conversion is suitable for direct tracking of dynamic capacitance changes. AC‐excited readout can provide the simplest circuit structure, whereas SC circuits provide an array‐compatible charge transfer interface, although both require careful control of parasitic coupling. Capacitance‐to‐time/frequency conversion using an RC oscillator or other oscillators simplifies digital acquisition but transfers the sensitivity to oscillator drift and parasitic capacitance. A CDC provides a direct digital output and avoids the need to design a separate ADC, but its usable performance depends on whether the sensor baseline capacitance and variation fit within the converter input range. Architecture selection for CS should therefore jointly consider resolution, parasitic capacitances, required conversion speed, and integration level.

Overall, the EQ category determines the basic conversion principle, whereas the dominant performance constraint determines the specific architecture and auxiliary techniques. The final readout system may combine several methods to form an integrated readout system, such as RTIA followed by FDA conditioning, CTIA combined with CDS, or bridge readout followed by programmable gain and calibration.

### 5.4 Low‐Noise Design

The SNR is one of the most important metrics for graphene‐based sensing systems. Low‐noise design therefore requires consideration of both device‐level noise originating from graphene sensors and circuit‐level noise introduced by the readout electronics. According to their spectral characteristics, the device‐level noise sources of graphene‐based sensors mainly include two types: broadband white noise and low‐frequency noise. Low‐frequency noise can be further divided into continuous 1/*f* noise and discrete Lorentzian components [222, 223].

According to the physical origin, broadband white noise mainly includes Johnson–Nyquist noise associated with the channel resistance of graphene‐based materials and graphene–metal contact resistance [224], and shot noise arising from stochastic charge transmission under finite‐bias nonequilibrium transport [225, 226]. For a two‐terminal graphene sensor, the Johnson–Nyquist noise can generally be expressed as [227]:

(14) $$S_{V,\;\mathrm{th}}=4k_{B}T\left(R_{\mathrm{ch}}+R_{c}\right)$$

(15) $$S_{I,\;\mathrm{th}}=\frac{4k_{B}T}{R_{\mathrm{ch}}+R_{c}}$$

where *S*_*V*, th_ and *S*_*I*, th_ are the voltage and current noise PSD, respectively, *k*_B_ is the Boltzmann constant, *R*_ch_ is the channel resistance, and *R*_c_ is the total contact resistance. In the high bias limit *e*|*V*|  ≫  *k*_B_ *T*, the shot‐noise current spectral density can be approximated as [225, 226]:

(16) $$S_{I,\;\mathrm{shot}}=2eF\left|I\right|$$

where *I* is the average current, and *F* is the Fano factor determined by the transmission probabilities of the conducting channels.

Broadband white noise can be mitigated through bandwidth limitation, integrating readout, low‐noise front‐end matching, and multi‐device averaging. Bandwidth limitation retains the signal frequency band while attenuating out‐of‐band noise, typically using a low‐pass filter for slowly varying sensor signals. Considering a flat noise PSD *S*_0_ and a normalized transfer function *H*(*f*) of the low‐pass filter, the output noise variance can be expressed as:

(17) $$\sigma_{n}^{2}=\int_{0}^{\infty }S_{0}\;{\left|H\left(f\right)\right|}^{2}df=S_{0}\;\mathrm{ENBW}$$

where ENBW denotes the equivalent noise bandwidth. Common analog implementations include passive RC low‐pass filters, Sallen–Key and multiple‐feedback active filters, and higher‐order Butterworth or Bessel low‐pass filters. As a practical example, Shah et al. processed the output of a graphene Hall sensor using a high‐pass filter with a cutoff frequency of 1 kHz, two cascaded THS4131C amplifiers, and a low‐pass anti‐aliasing filter with a cutoff frequency of 100 kHz. The acquired signal was subsequently digitally band‐pass filtered over 1–10 kHz and integrated, and the processed signals exhibited an average SNR of 20–23 dB [228]. Integrating readout uses a capacitor to accumulate the input charge over a defined integration interval *T*_0_. For a CTIA, the output can be expressed as:

(18) $$V_{\mathrm{out}}=V_{\mathrm{reset}}\;-\;\frac{1}{C_{\mathrm{int}}}\int_{0}^{T_{0}}i_{\mathrm{in}}\left(t\right)dt$$

where *C*_int_ is the integration capacitor, *V*_reset_ is the initial output voltage after reset, and *i*_in_ is the input current. For a constant input current, the SNR ideally improves as $\sqrt{T_{0}}$ [229]. Increasing the integration time therefore improves sensitivity but reduces temporal resolution and may lead to saturation when the accumulated charge exceeds the available output swing. Practical limitations also include *kT*/*C* reset noise, charge injection, and clock feedthrough introduced by the reset switch. Low‐noise front‐end matching aims to minimize the noise added by the readout electronics. For a voltage‐output sensor represented by a source impedance *Z*_s_(*f*), the simplified total input voltage noise PSD can be written as:

(19) vn,total2f≈4kBTReZsf+vn2f+in2fZsf2+vn,fb2f

where *v*_n_(*f*) and *i*_n_(*f*) are the input voltage‐ and current‐noise densities of the front‐end amplifier, respectively, and *v*_n, fb_(*f*) represents the equivalent input noise of the feedback network. Multi‐device averaging relies on approximately matched sensor responses and statistically uncorrelated noise among different devices. For *N* devices with identical sensitivities, averaging preserves the signal while reducing the uncorrelated noise amplitude by a factor of $\sqrt{N}$. Shetty et al. connected two epigraphene Hall sensors in parallel and found that, at 20 kHz, the voltage noise PSD of the parallel configuration was approximately $1/\sqrt{2}$ of that of a single device, resulting in improvement of SNR [230].

Low‐frequency noise can generally be represented by two spectral components: a continuous 1/*f* background and Lorentzian components. Continuous 1/*f* noise is commonly attributed to fluctuations in the carrier number and carrier mobility [223, 231]. It can be described phenomenologically as:

(20) $$S_{X}\left(f\right)=\frac{A_{X}}{f^{\gamma }}$$

where *X* can denote current, voltage, resistance, or conductance, *A_X_* is the corresponding noise‐amplitude prefactor, and γ is typically close to 1. In graphene‐based sensors, such low‐frequency fluctuations can originate from several device and interface processes, including trapping, scattering, adsorption‐related fluctuations, and contact effects [54, 222, 232, 233, 234, 235]. From a readout perspective, the resulting 1/*f* noise is particularly important for low‐frequency and quasi‐DC sensing because it can directly limit the achievable SNR. When discrete switching processes dominate the low‐frequency fluctuations, the measured signal may exhibit random telegraph noise (RTN) [236]. In the frequency domain, the one‐sided PSD of a single RTN component has a Lorentzian form:

(21) $$S_{L}\left(f\right)=\frac{4\sigma^{2}\tau }{1+{\left(2\pi f\tau \right)}^{2}}$$

where σ^2^ is the variance contributed by the fluctuator and τ is the correlation time determined by the transition rates between the two states. The spectrum exhibits an approximately frequency‐independent plateau at *f*  ≪  *f*_c_, rolls off near the corner frequency *f*_c_  =  1/(2πτ), and decreases approximately as 1/*f* ^2^ at *f*  ≫  *f*_c_. Such discrete low‐frequency fluctuations can introduce time‐dependent baseline variations and further degrade the repeatability and SNR of slowly varying sensing signals.

Low‐frequency noise can be mitigated through modulation and synchronous demodulation, bias point optimization, the selection of front‐end circuits with low input 1/*f* noise, and multi‐device averaging. In modulation and synchronous demodulation, the stimulus, gate voltage, bias, or sensor conversion coefficient is periodically modulated so that the target signal is translated from near DC to the modulation frequency *f*_m_. For example, under the on‐off optical modulation commonly used in photoelectric sensing, the sensor output can be represented as

(22) $$X_{\mathrm{out}}\left(t\right)=\frac{X_{\mathrm{on}}}{2}\;\left[1+\mathrm{sgn}\left(\sin2\pi f_{m}t\right)\right]$$

where *X*_on_ is the sensor output in the illuminated state. This expression describes a square‐wave modulation between 0 and *X*_on_ with a 50% duty cycle. An LIA then performs phase‐sensitive demodulation and narrowband detection around *f*_m_, where the 1/*f* noise is generally lower. Such modulation and synchronous demodulation schemes have been employed for weak‐signal readout in graphene‐based photoelectric sensors [237], magnetic sensors [135], and molecular sensors [238]. The basic idea of bias point optimization is that the 1/*f* noise of graphene devices depends on the gate voltage *V*_G_, drain‐to‐source voltage *V*_DS_, and bias current *I*_B_ [239]. Therefore, programmable control of the *V*_G_, *V*_DS_, and *I*_B_ can be used to select an operating point that provides an optimal trade‐off between sensor response and noise, rather than simply maximizing the electrical response. For example, Fu et al. demonstrated ambipolar biosensing near the CNP of graphene. Operating near the CNP markedly reduced the 1/*f* noise without compromising the sensing response, thereby improving the SNR compared with conventional operation at the maximum‐transconductance point [48]. Schaefer et al. characterized the Hall response and voltage noise of ultraclean hBN‐encapsulated graphene Hall sensors and found the optimum operating point represented a trade‐off between approaching the CNP to increase the Hall coefficient and moving away from the CNP to reduce the voltage noise [33]. The purpose of selecting front‐end circuits with low input 1/*f* noise is to prevent the low‐frequency noise introduced by the amplifiers and bias circuitry from masking the weak signals generated by graphene devices. For VSS, the output can be directly amplified using a voltage preamplifier with low 1/*f* noise. For CSS, a front‐end TIA with low 1/*f* noise can be used to convert the sensor current into a voltage. Furthermore, multi‐device averaging can also suppress device‐specific, uncorrelated 1/*f* noise components.

### 5.5 Mitigation of Nonidealities in Graphene‐Based Sensors

This section discusses major nonidealities affecting graphene‐based sensing systems, including environmental instability, device‐to‐device variability, screening effect, and non‐specific binding, and examines circuit‐ and system‐level strategies for mitigating these issues, as summarized in Table 4.

**TABLE 4:** Circuit‐ and system‐level strategies for mitigating major nonidealities in graphene‐based sensing systems.

Nonideality	Observable consequence	Strategy	Representative implementation
Environmental instability	Baseline drift; transfer‐curve hysteresis; CNP shift.	Baseline correction	Profile‐matching calibration [55]
		Adaptive compensation	Model‐based temperature and humidity compensation [246]
		Differential readout	Measurement‐reference differential readout [247]
		Operating‐point stabilization	DMF feedback [37]
		AC excitation and phase‐based readout	AC phase‐lag readout [248]
Device‐to‐device variability	Dispersion in baseline levels, transfer curves, responsivity.	Statistical model‐based calibration and inference	Array inference by random forest [55]
		Outlier detection and channel masking	Impedance‐ and noise‐based channel preselection [42]
Screening effect	Attenuation of electrostatic gating; reduced sensitivity and DC response.	Nonequilibrium high‐frequency readout	AC‐mode heterodyne readout [258]
Non‐specific binding	Responses to nontarget molecules and elevated blank signal.	Comparative multichannel readout	Differential sensing‐reference array readout [263]

The environmental instability of graphene‐based sensors largely arises from the atomically thin and exposed nature of graphene, which makes its electrical properties highly sensitive to ambient conditions. Major contributors include adsorbate‐induced doping by water, oxygen, and other environmental species [49, 50, 240, 241], temperature‐dependent changes in carrier transport and device resistance [242], as well as measurement‐induced drift caused by repeated gate‐voltage sweeps and sustained bias stress [243]. These effects can manifest as baseline drift, transfer‐curve hysteresis, and time‐dependent shifts in the CNP [49, 244]. Circuit‐ and system‐level approaches for mitigating environmental instability include baseline correction, adaptive compensation, differential readout, operating‐point stabilization, and modulation and synchronous demodulation. Baseline correction first measures the device output under a known zero‐input or reference condition and subtracts this baseline from subsequent measurements. If the baseline continues to drift, its estimate can be updated periodically. However, drift occurring between calibration events remains uncompensated, and slowly varying target signals may be partially removed if they cannot be reliably distinguished from baseline drift [245]. As a more advanced baseline correction example, Xue et al. developed an integrated ion‐sensing platform containing a 16 × 16 GFET array. Their profile‐matching calibration method used a single calibration solution of known concentration to obtain the current distributions of the array at different gate voltages. The current distribution measured for an unknown sample was then matched to the calibration data through least‐squares fitting, allowing the equivalent gate‐voltage shift and ion concentration to be estimated. The calculated concentrations exhibited an *R* ^2^ of 0.996 relative to the nominal concentrations. The averaged sensor sensitivity also showed negligible drift over a six‐month period [55]. Adaptive compensation uses independent temperature, humidity, or other auxiliary sensors to monitor environmental variables in real time and estimate their contributions to the graphene‐sensor output. The compensated signal can be expressed as:

(23) $$X_{\mathrm{comp}}\;\left(t\right)=X_{\mathrm{raw}}\;\left(t\right)\;-\;\hat{d}\;\left[T\left(t\right),RH\left(t\right),\text{…}\right]$$

where *X*_raw_(*t*) and *X*_comp_(*t*) are the raw and compensated outputs, respectively, *RH*(*t*) is the relative humidity, and $\hat{d}$ represents the estimated environmental contribution. This approach enables continuous online correction, but generally requires a device‐specific model for calibration. Its performance may also be limited by mismatched response times between the graphene and auxiliary sensors. Park et al. developed a drone‐mountable gas‐sensing platform comprising three graphene chemiresistors, a commercial temperature‐humidity sensor, a 32‐bit ADC, and an MCU. A device‐specific model related the normalized graphene resistance change to temperature and humidity was established, and the predicted environmental contribution was subtracted from the raw signal. In outdoor tests, the average standard deviation of the three graphene‐sensor signals decreased from 0.53% to 0.08% [246]. Differential readout employs a sensing channel and a reference channel with different sensitivities to the target but similar responses to environmental disturbances and drift. Subtracting the two outputs preserves their differential target response while suppressing correlated common‐mode disturbances. Its effectiveness is limited by mismatch between the two channels and by disturbances that are uncorrelated or coupled differently to them. For example, Li et al. developed a differential GFET affinity‐sensing system comprising a target‐sensitive measurement unit and a reference unit designed to respond to nonspecific interferences. By differentially processing the two channel outputs, common‐mode disturbances arising from environmental variations could be suppressed. The system maintained consistent detection of 17β‐estradiol in the presence and absence of pH variations, demonstrating the effectiveness of differential readout for improving measurement robustness under changing environmental conditions [247]. Repeated gate‐voltage sweeps can perturb trap occupancy, ionic distributions, and interfacial charge at the dielectric and graphene–electrolyte interfaces, thereby increasing hysteresis, signal drift, and operational instability. Operating‐point stabilization mitigates these effects by either fixing the device at a selected bias point or using closed‐loop feedback to maintain a predefined channel state, thereby avoiding drift introduced by repeated gate sweeps. Kammarchedu et al. developed a dual‐gate GFET integrating a local HfO_2_ back gate with an electrolyte top gate and employed a closed‐loop controller to adjust the back‐gate voltage in real time. In the differential mode fixed (DMF) configuration, the feedback loop maintained a predefined channel current while converting analyte‐induced surface‐potential changes into amplified back‐gate voltage shifts. During volatile organic compounds (VOC) sensing, the DMF mode exhibited a drift rate of 2.65%/h, more than 15 times lower than the 38.91%/h obtained in the continuously swept back‐gate mode [37]. Furthermore, AC excitation and phase‐based readout can reduce the influence of slowly varying baseline variations by using frequency‐ or phase‐domain features instead of conventional DC resistance changes. Liu et al. applied an AC gate voltage to a CVD GFET and used the phase lag of the channel response relative to the gate excitation as the sensing signal. The resulting phase responses enabled the detection of water, methanol, and ethanol vapors. Compared with conventional DC resistance readout, the AC phase signals exhibited minimal baseline drift and recovery times at least tenfold shorter [248].

Device‐to‐device variability refers to the substantial differences in baseline electrical characteristics and sensing responses among graphene devices fabricated using nominally identical materials, structures, and processes. Its major origins include growth‐related spatial nonuniformities in graphene [249, 250], transfer‐induced damage [251, 252], and spatial nonuniformities in the substrate [253, 254]. Circuit‐ and system‐level approaches for mitigating device‐to‐device variability mainly include statistical model‐based calibration and inference, and outlier detection and channel masking. Statistical model‐based calibration and inference characterize the transfer curves and sensing responses of individual devices and then use normalization, statistical regression, or machine‐learning models to map the diverse device outputs to the target quantity. For example, Xue et al. extracted multidimensional features from the measured *I*–*V* characteristics of individual devices, including the Dirac‐point voltage and the maximum and minimum transconductance, and used these features to train a random forest model. The model achieved an ion‐type classification accuracy of 97.6%. Compared with models using only 25 devices, the use of more than 200 devices improved the accuracy of some classification tasks by more than 20 percentage points [55]. Outlier detection and channel masking first characterize individual channels and assign quality labels according to predefined electrical or noise‐based criteria. Only qualified channels are retained for subsequent readout, thereby preventing severely abnormal devices from biasing the array output. Maity et al. identified nonideal GFETs using impedance and noise measurements and excluded these channels. For the same Pb^2^^+^ concentration of 5 ppb, the response distribution after device preselection was substantially narrower than that obtained without preselection [42].

In liquid‐gated GFET biosensors, the screening effect arises from the redistribution of mobile ions in response to the electrostatic field generated by charged biomolecules, thereby attenuating the electrostatic potential reaching the graphene channel. Under the linearized Debye–Hückel approximation, this electrostatic potential decays approximately exponentially with the distance from the charged target and is strongly influenced by the Debye screening length [255, 256]. The main consequences include attenuation of target‐induced electrostatic gating [257], reduced sensitivity with increasing ionic strength, and suppression of DC sensing response [258]. At the circuit and system levels, a representative mitigation strategy is nonequilibrium high‐frequency readout. Under DC or low‐frequency excitation, mobile ions have sufficient time to redistribute and establish a near‐equilibrium electrical double layer, thereby screening the electrostatic field of the target. When the excitation frequency exceeds the relevant ionic relaxation, the ions cannot fully follow the rapidly varying electric field, and the ionic atmosphere cannot completely re‐equilibrate during each cycle. Consequently, frequency‐dependent molecular responses can remain detectable despite strong equilibrium screening [259]. For a GFET with nonlinear transfer characteristics, an amplitude‐modulated carrier can be applied as [260]:

(24) $$V_{\mathrm{RF}}\;\left(t\right)=V_{c}\left(1+m\cos\omega_{m}t\right)\cos\omega_{c}t$$

where *V*_c_ and ω_c_ are the carrier amplitude and angular frequency, respectively, and *m* and ω_m_ are the modulation depth and modulation angular frequency. The carrier and its sidebands undergo self‐mixing through the nonlinear GFET response, generating a down‐converted current component at ω_m_, which can subsequently be extracted using an LIA. Sarker et al. employed this AC‐mode heterodyne GFET architecture to detect neuropeptide Y (NPY) in artificial sweat at physiologically relevant ionic concentrations. In contrast to the DC‐mode response, which was strongly limited by ionic screening, the AC‐mode response increased with electrolyte ionic strength and could be further optimized by tuning the carrier frequency. The sensor achieved a detection limit of 2 am and a dynamic range spanning approximately ten orders of magnitude [258].

Non‐specific binding refers to the unintended adsorption or binding of nontarget molecules to the graphene surface. It can arise from the strong non‐specific adsorption of proteins onto bare or insufficiently passivated graphene [261], competitive occupation of surface sites by abundant background constituents [262, 263], and incomplete receptor coverage or ineffective blocking of unfunctionalized surface regions [264]. It commonly manifests as responses to nontarget molecules and elevated blank signals. At the circuit and system levels, the influence of non‐specific binding can be reduced through comparative multichannel readout. Comparative multichannel readout mitigates non‐specific binding by comparing the responses of sensing channels with those of reference channels exposed to the same sample. Differential readout is the simplest two‐channel implementation for this method. For example, Yin et al. developed a 12‐device GFET array comprising six sensing channels and six antibody‐free reference channels exposed simultaneously to the same plasma sample. The differential Dirac‐voltage shift between the two channel groups suppressed common background responses and enabled pancreatic ductal adenocarcinoma and healthy plasma samples to be distinguished with *P* < 0.0001 [263].

## 6 Summary and Outlook

Graphene‐based materials, owing to their distinctive zero‐bandgap Dirac‐cone energy band structure, exhibit exceptional electrical, thermal, optical and mechanical properties. These characteristics together make graphene‐based materials attractive material platforms for diverse sensing domains, including optoelectronic, molecular, mechanical, magnetic and thermal sensing. As graphene‐based sensors provide diverse electrical outputs and the sensing information is often encoded as changes in these electrical quantities, appropriate readout circuits are essential for translating device‐level transduction into stable, processable system‐level signals. The field has therefore moved toward a sensor‐electronics co‐design mindset, where device physics and interface circuitry are treated as a coupled system rather than as independent modules. In this review, we propose a unifying PS–EQ–RF framework to connect physical stimuli, electrical quantities, and readout features, thereby providing general design principles that bridge sensing phenomena with practical circuit implementation. Based on this framework, we present a systematic review of graphene‐based sensors classified by application scenarios and output signal types. We further summarize representative readout methods and implementation paradigms tailored to each electrical output category, and provide system‐level co‐design guidelines for the graphene‐based sensing systems.

**FIGURE 17:**
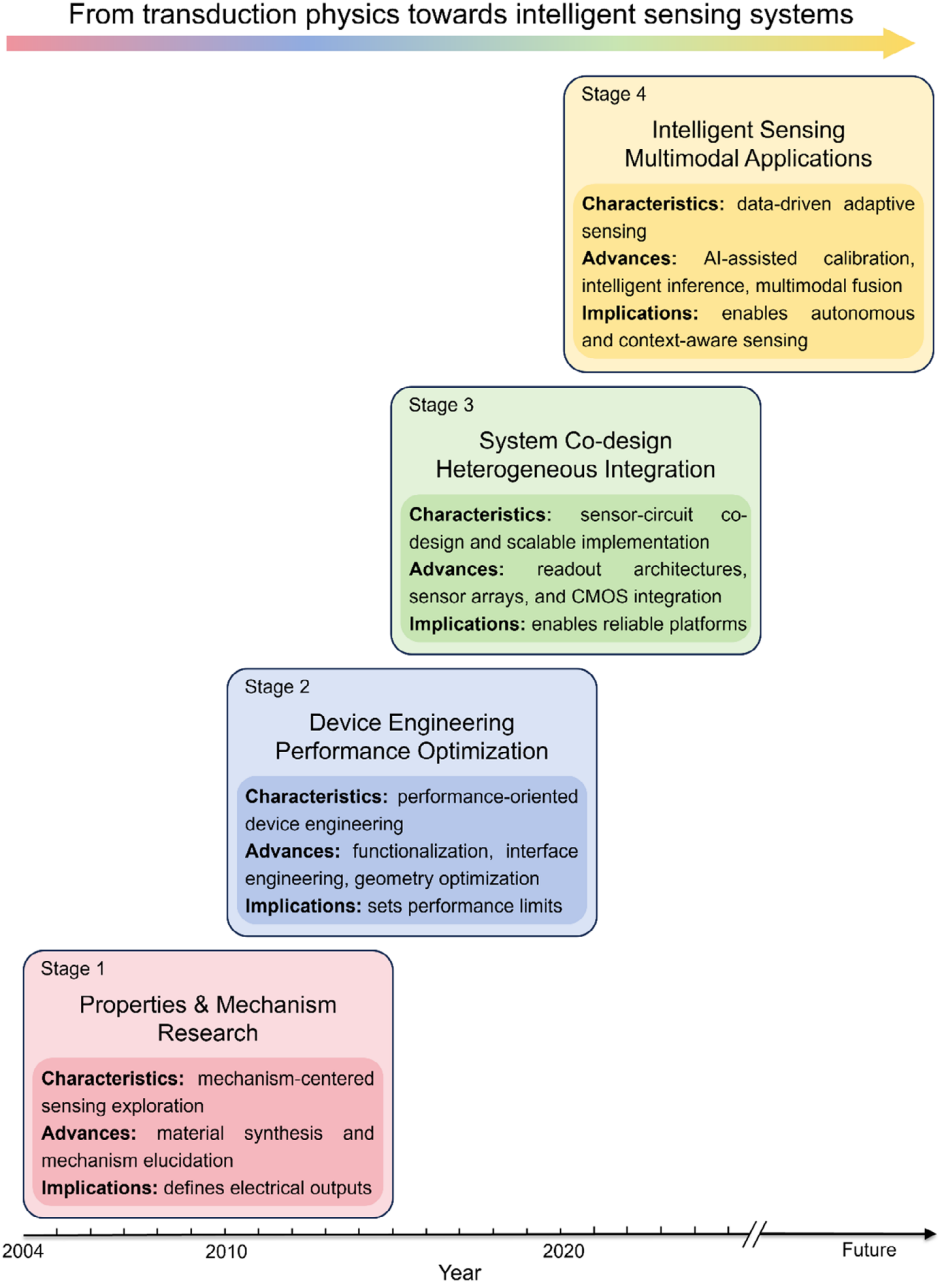
Four‐stage evolution framework for graphene‐based sensing systems, highlighting the defining characteristics, technological advances, and system‐level implications of each stage.

From a system‐integration perspective, the evolution of graphene sensing systems can be viewed as four stages (Figure 17). Stage 1 (2004‐2015) focused on exploring the intrinsic properties of graphene and verifying fundamental sensing mechanisms. Stage 2 (2009‐2020) highlighted advances in micro‐and nanofabrication techniques and performance enhancement of graphene‐based devices. Since 2015, stage 3 has targeted the co‐design of sensors and readout circuits, with particular attention to challenges in graphene‐CMOS heterogeneous integration. More recently, stage 4 (2020‐present) has shifted toward multimodal sensing and intelligent sensing systems driven by rapid advances in artificial intelligence (AI). These stages are not strictly sequential and may overlap, as device‐level advances and system‐level co‐design progressed in parallel.

In particular, with the continued advancement of AI, its application to different functions of graphene‐based sensing systems has emerged as an important research direction. These applications can be broadly distinguished into two levels: AI‐assisted data analysis, which uses AI to process and interpret acquired sensor data, and AI‐enabled readout architectures, in which AI is incorporated into the sensing acquisition or control process. In AI‐assisted data analysis, representative functions include drift compensation, adaptive calibration, fault detection and error correction, and multimodal sensing integration. For drift compensation, AI models can learn slowly varying baseline patterns from historical time‐series data, reference‐channel outputs, and environmental variables, and can distinguish or compensate for these variations when estimating the target response. For example, Capman et al. used graphene varactor sensors to detect three types of volatile organic compounds (VOCs) at different oxygen concentrations and employed a long short‐term memory network to analyze the complete time‐series responses. The model was still able to identify the VOC types and concentrations, demonstrating the feasibility of AI‐enabled drift‐robust inference and providing a methodological basis for future quantitative baseline estimation and compensation [265]. For adaptive calibration, AI can utilize array redundancy, initial device characteristics, and newly acquired calibration data to learn the offset, gain, and responsivity, thereby adapting to device‐to‐device variations and changes in operating conditions. As an early data‐driven example, Zhang et al. extracted features from the transfer and output characteristics of graphene EGFET Ca^2^^+^ sensors and trained regression models to directly estimate ion concentrations. The random‐forest model using transfer‐curve features achieved the best performance and reduced the dependence of concentration estimation on any single gate‐bias point, thereby mitigating the influence of gate‐bias‐dependent variations in sensitivity and response range [266]. For fault detection and error correction, AI can integrate multidimensional features, including DC characteristics, impedance, noise spectra, and temporal response, to identify channels that deviate from the normal device distribution and subsequently correct their outputs. As a representative array‐level quality‐control example, Maity et al. used impedance and noise measurements to identify faulty devices that could not be reliably distinguished using conventional DC characteristics alone in large‐scale GFET arrays. After excluding the abnormal devices, they employed machine learning to classify and quantify the sensing responses and demonstrated real‐time simultaneous monitoring of heavy‐metal ions and bacteria in flowing tap water [42]. For multimodal sensing integration, AI can fuse signals from different sensing modalities or feature domains and generate joint estimates or classifications. For example, Yang et al. developed a single‐component multimodal sensor based on the moisture‐electric response of graphene oxide and used machine learning to interpret and decouple its multidimensional outputs, enabling the simultaneous monitoring of temperature, humidity, pressure, and light intensity [267].

In contrast, AI‐enabled readout architectures are emerging along several directions, including AI‐assisted readout co‐design, in‐sensor computing, and adaptive power management. For AI‐assisted readout co‐design, machine‐learning models can be incorporated into the readout mapping between electrically encoded sensor features and the target quantities, allowing the generation of readout features and subsequent inference to be considered together. For example, Hajizadegan et al. proposed a multi‐GFET harmonic sensing architecture in which analyte information was encoded into multiple harmonic components generated by the GFET sensors, and a neural network served as the readout model to estimate multiple analyte levels from these harmonics. The dependence of inference accuracy on the number of available harmonics illustrated the potential of jointly considering the design of the multi‐GFET sensing architecture and machine‐learning‐based readout [268]. For in‐sensor computing, graphene‐based devices with dynamic or memory‐dependent responses can provide physical states that are directly exploited by AI algorithms for information processing, allowing sensing and computation to be more tightly integrated. For example, Zhang et al. developed a GO ionic sensory memristive device whose ion‐dynamic responses served as a physical reservoir for reservoir‐computing‐based flavor classification, demonstrating the integration of chemical sensing and AI‐based neuromorphic processing within a single device platform [269]. For adaptive power management, AI may dynamically adjust the sampling rate, active‐channel configuration, bias conditions, and communication duty cycle according to signal activity, task requirements, battery level, and available harvested energy, thereby reducing energy consumption. As a general wireless‐sensor example, Heo et al. proposed a predictive energy‐aware adaptive‐sampling method based on deep reinforcement learning that selected the sampling frequency according to the recent history of harvested power and battery level [270]. This method illustrates a transferable system‐level strategy for the future energy management of autonomous graphene sensing systems. While such examples are still relatively few, they highlight the emerging potential of integrating AI more closely with readout and system design in graphene‐based sensing platforms.

While current advances have established a solid technical foundation, graphene‐based sensing systems still face critical challenges [271, 272]. The primary bottleneck in graphene sensing is no longer merely the demonstration of high sensitivity in individual devices, but the achievement of performance that is reproducible, calibratable, stable over extended operation, manufacturable at scale, and compatible with system‐level integration under realistic operating conditions. Despite substantial progress, the translation of graphene‐based sensing systems from laboratory prototypes to practical applications remains constrained by four major categories: scientific, technological, manufacturing, and integration challenges.

At the scientific level, the central challenge is to establish a mechanistic understanding of graphene sensing processes that enables their interpretation, separation, and prediction. Two major bottlenecks remain. First, target‐induced responses and nonideal contributions, including cross‐sensitivity, non‐specific adsorption, and environmental drift, can produce similar changes, while multiple transduction mechanisms may operate simultaneously. Consequently, an observed electrical output alone is often insufficient to identify its underlying physical origin [50, 273, 274]. In the short term, priorities should include controlled measurements that provide complementary electrical observables under different bias or environmental conditions, together with reference‐assisted measurements where appropriate, to help distinguish target‐induced responses from nonideal contributions. These measurements should also be complemented by physically interpretable semi‐empirical models that relate electrical outputs to operating bias, time, physical stimuli, environmental variables, and major noise contributions [275, 276]. In the long term, the field should move toward modality‐specific physics‐informed models that predict electrical responses across different operating and environmental conditions by linking physical stimuli, sensing mechanisms, device states, electrical outputs, and major noise sources. Combined with orthogonal electrical observables, such models could provide a quantitative basis for mechanism‐level signal separation and distinguish target‐induced responses from reversible environmental perturbations and irreversible device aging [277]. Second, key figures of merit of the sensing system, including sensitivity, noise floor, response time or bandwidth, dynamic range, and stability, are interdependent and are jointly governed by the sensing mechanism, material and interface properties, device geometry, operating point, and readout conditions. Improving one metric may therefore compromise another, and mechanism‐specific strategies are still required to optimize overall system performance rather than any single metric in isolation [278, 279]. In the short term, these coupled trade‐offs should be quantitatively characterized under application‐relevant operating conditions to identify suitable operating regions and guide the joint selection of sensor operating points and readout parameters. In the long term, multi‐objective co‐design frameworks should be developed to quantify how sensing conditions and readout parameters jointly affect system‐level figures of merit, thereby enabling application‐specific sensor‐readout optimization rather than the isolated maximization of individual performance metrics.

At the technological level, the central challenge is to achieve scalable, adaptive, and reliable readout and control of multi‐device graphene sensor arrays. Two major bottlenecks remain. First, device‐to‐device variations in the Dirac point, transconductance, contact resistance, noise, and sensing response tend to translate into channel‐dependent baselines, optimal bias points, gain, dynamic range, and calibration requirements, thereby complicating uniform array operation [280]. In the short term, programmable and channel‐adaptive biasing and readout [56], reference‐assisted calibration [263], and low‐overhead digital calibration [55] provide practical routes compensating channel‐to‐channel variations and maintaining appropriate operating conditions across the array. In the long term, self‐characterizing and self‐calibrating arrays should be developed to automatically identify channel variations and faulty devices, adjust operating points, and maintain consistent array‐level performance. Second, increasing the channel count introduces coupled system‐level trade‐offs among interconnect and multiplexing complexity, readout bandwidth, acquisition time, power and area, and data throughput [281]. In the short term, these scaling trade‐offs should be quantitatively evaluated according to application requirements, while multiplexing ratio, channel activation, sampling rate, and readout bandwidth are jointly configured to balance measurement throughput and hardware overhead. In the long term, scalable and reconfigurable readout architectures should enable these resources to be dynamically allocated across sensor channels according to signal characteristics and application requirements, providing a path toward higher‐channel‐count graphene sensing systems without proportionally increasing readout complexity, power, and data throughput.

At the manufacturing level, the central challenge is end‐to‐end functional yield in the production process. Two major bottlenecks remain. First, non‐uniformity in wafer‐scale graphene and variations introduced during subsequent fabrication processes can propagate into device‐to‐device variations in electrical and sensing characteristics, thereby reducing usable device yield and increasing the calibration burden at the array and system levels [282, 283, 284, 285, 286]. In the short term, wafer‐level statistical process control, in‐line metrology, and electrical screening should be used to quantify and control these variations, with acceptance criteria based on distributions of key electrical and sensing parameters rather than material quality alone [56, 287]. In the long term, manufacturing specifications should be coordinated with the tolerance and calibration capability of the readout system, enabling reproducible process platforms qualified across wafers and fabrication lots [288, 289, 290, 291]. Second, different graphene sensing applications impose different requirements on device fabrication, functionalization, passivation, and packaging, making a single manufacturing route unsuitable for all sensor modalities [292]. In the short term, application‐specific process flows and process windows should therefore incorporate these steps into the complete manufacturing sequence rather than treating them as independent post‐fabrication operations. In the long term, application‐specific manufacturing qualification criteria should be established to define acceptable process‐induced variations in electrical and sensing performance, together with packaging reliability and end‐to‐end functional yield requirements.

At the integration level, the central challenge lies in achieving scalable and reliable heterogeneous integration of graphene sensors with CMOS readout and processing circuits. Two major bottlenecks remain. First, graphene growth, transfer, patterning, and functionalization processes are not yet fully compatible with the thermal budgets and contamination‐control requirements of CMOS technology [163, 293, 294]. In the short term, application‐specific post‐CMOS or hybrid integration approaches provide practical routes for separating graphene‐sensitive processing steps from standard CMOS fabrication. In the long term, reproducible integration flows should be established with quantified limits on thermal budget, contamination, interface stability, and packaging reliability across complete sensor‐CMOS assemblies. Second, electrical interconnections can introduce parasitic resistance and capacitance, noise coupling, and signal attenuation, while graphene sensing devices, readout circuits, and downstream processing modules are still often optimized separately. Together, these factors can limit system‐level performance even when the individual components perform well [294, 295, 296]. In the short term, modular sensor‐PCB/ASIC platforms can be used to jointly evaluate sensor‐readout interfaces, parasitic effects, noise, dynamic range, and calibration strategies before tighter integration [263, 297]. In the long term, interconnects, front‐end circuits, and near‐sensor processing should be co‐designed under explicit constraints on noise, bandwidth, dynamic range, power, and packaging, with the degree of physical integration selected according to the target application [298].

In conclusion, with ongoing breakthroughs in manufacturing techniques, heterogeneous integration and AI‐driven multimodal sensing, graphene‐based sensing systems are poised to progress rapidly from laboratory prototypes to practical deployment. Under this trend, graphene‐enabled platforms are well positioned to support new capabilities in intelligent optoelectronic sensing, flexible electronic skin and implantable biosensors, and they are expected to deliver innovative technological impacts in the near future.

## Author Contributions

**Jinduo Zhang**: conceptualization, writing – original draft, writing – review and editing, visualization, investigation, methodology. **Meng Chen**: conceptualization, writing – original draft, methodology, writing – review and editing, funding acquisition. **Yingxin Wang**: conceptualization, funding acquisition, writing – original draft, writing – review and editing, project administration, supervision, resources, methodology. **Guanchen Li**: investigation, visualization, methodology. **Ruifeng Liu**: writing – original draft, methodology, visualization. **Ziran Zhao**: conceptualization, funding acquisition, writing – original draft, writing – review and editing, visualization, project administration, methodology, supervision, resources.

## Funding

This research was supported by the National Natural Science Foundation of China (Nos. 62327804, 62375149); Beijing Nova Program (Nos. 20240484648, 20240484671); and the National Key R&D Program of China (No. 2023YFF0715000).

## Conflicts of Interest

The authors declare no conflicts of interest.

## Data Availability

Data sharing not applicable to this article as no datasets were generated or analyzed during the current study.
